# Mucosal kinase activity and inflammatory profiles in inflammatory bowel disease, and in relation to tofacitinib response

**DOI:** 10.1093/ecco-jcc/jjaf174

**Published:** 2025-09-23

**Authors:** Eelco C Brand, Britt Roosenboom, Lisanne Lutter, Bea Malvar Fernandez, Savithri Rangarajan, Elly van Koolwijk, Sara van Gennep, Geert R D’Haens, Ellen G van Lochem, Carmen S Horjus Talabur Horje, Kris A Reedquist, Femke van Wijk, Bas Oldenburg

**Affiliations:** Department of Gastroenterology and Hepatology, University Medical Center Utrecht, Utrecht University, Utrecht, The Netherlands; Center for Translational Immunology, University Medical Center Utrecht, Utrecht University, Utrecht, The Netherlands; Crohn & Colitis Center Rijnstate, Department of Gastroenterology and Hepatology, Rijnstate Hospital, Arnhem, The Netherlands; Department of Gastroenterology and Hepatology, University Medical Center Utrecht, Utrecht University, Utrecht, The Netherlands; Center for Translational Immunology, University Medical Center Utrecht, Utrecht University, Utrecht, The Netherlands; Galicia Sur Health Research Institute, Rheumatology and Immune-mediated Diseases Group, Vigo, Spain; previously affiliated with the Center for Translational Immunology, University Medical Center Utrecht, Utrecht University, Utrecht, The Netherlands; PamGene International B.V., ‘s-Hertogenbosch, The Netherlands; Department of Microbiology and Immunology, Rijnstate Hospital, Arnhem, The Netherlands; Department of Gastroenterology and Hepatology, Amsterdam UMC, Amsterdam, The Netherlands; Department of Gastroenterology and Hepatology, Amsterdam UMC, Amsterdam, The Netherlands; Department of Microbiology and Immunology, Rijnstate Hospital, Arnhem, The Netherlands; Crohn & Colitis Center Rijnstate, Department of Gastroenterology and Hepatology, Rijnstate Hospital, Arnhem, The Netherlands; Independent researcher; previously affiliated with the Center for Translational Immunology, University Medical Center Utrecht, Utrecht University, Utrecht, The Netherlands; Center for Translational Immunology, University Medical Center Utrecht, Utrecht University, Utrecht, The Netherlands; Department of Gastroenterology and Hepatology, University Medical Center Utrecht, Utrecht University, Utrecht, The Netherlands

**Keywords:** PamGene, Luminex, response prediction

## Abstract

**Background and Aims:**

Not all patients, as with other inflammatory bowel disease (IBD) treatments, respond to modulation of kinase activity. To improve the precision of therapeutic interventions, a better understanding of the mucosal inflammatory environment is essential. This study investigates mucosal kinase activity and cytokine/chemokine profiles in IBD and in relation to tofacitinib response.

**Methods:**

Paired inflamed and non-inflamed colonic biopsies were collected from patients with Crohn’s disease (CD, *n* = 16), ulcerative colitis (UC, *n* = 16), and non-IBD controls (*n* = 4) to assess IBD-associated kinase activity and cytokine/chemokine profiles. Additionally, colonic samples were collected from UC patients before the start of tofacitinib treatment (cohort 1, *n* = 12) and both before and after 8 weeks of treatment (cohort 2, *n* = 16), to assess tofacitinib response-related kinase activity profiles.

**Results:**

The kinase activity profiles exhibited significant differences between inflamed and non-inflamed mucosa, with more pronounced alterations observed in UC compared to CD. The increase in kinase activity was most pronounced in the tyrosine kinase families. Responders to tofacitinib demonstrated higher baseline mucosal kinase activity, although only two predicted kinases (DCLK1 and ATR) were consistently identified. In responders, mucosal kinase activity significantly decreased after 8 weeks of treatment.

**Conclusion:**

Mucosal kinase activity profiles are associated with inflammation in IBD, with distinct differences between UC and CD. Baseline kinase activity appears to predict response to tofacitinib, with a marked reduction in kinase activity observed after 8 weeks of treatment in responders. These findings highlight the potential of kinase activity profiling in optimizing therapeutic strategies for IBD.

## 1. Introduction

Inflammatory bowel disease (IBD), comprising Crohn’s disease (CD) and ulcerative colitis (UC), are chronic relapsing–remitting inflammatory diseases of the gastrointestinal tract.^1^^,^^2^ The therapeutic armamentarium for IBD has rapidly expanded in the past decade, and now includes therapies targeting inflammatory cytokines (eg, tumor necrosis factor-α [anti-TNFα], interleukin-12 [IL12] and/or IL23), leukocyte migration (via integrin-α4β7 or the sphingosine-1-phosphate receptor), and kinase signaling (ie, the Janus kinase [JAK] family).^3^^,^^4^ Current medical treatments, however, have hit the therapeutic ceiling and are only effective in roughly 30%-60% of patients.^5^ To improve outcomes and enable personalized medicine we need to better understand the inflammatory environment and thus the pathophysiology of IBD.

JAK inhibitors are a relatively novel class of drugs in IBD, including tofacitinib (a pan-JAK inhibitor, predominantly inhibiting JAK1/JAK3), filgotinib (a JAK1 inhibitor), and upadacitinib (a JAK1 inhibitor). JAK-signal transducer and activator of transcription (STAT) signaling is a universally expressed intracellular signal transduction pathway that is involved in a range of biological processes, thereby constituting an obvious target for modulation of the dysregulated inflammation in IBD. In addition, genome-wide association studies have identified several variants in JAK2, tyrosine kinase 2 (TYK2), STAT1, STAT3, and STAT4 loci associated with IBD.^6^ The four JAKs (JAK1, JAK2, JAK3, and TYK2) are activated following the extracellular binding of cytokines to their cognate transmembrane receptors. Subsequently, STAT molecules are phosphorylated, dimerized, and translocated to the nucleus, resulting in gene transcription regulation.^7^ Because of its central role in a pleiotropic range of systems, the outcomes of inhibition of JAK–STAT signaling in patients with a heterogeneous disease, such as IBD, can be unpredictable. Furthermore, the inflammatory milieu in IBD is certainly more complicated than the JAK–STAT pathway alone. A better understanding of the local kinase activity and cytokine/chemokine production in IBD in general and related to response of treatment might aid in the pursuit of personalized medicine to exert maximal therapeutic efficacy.

We therefore aimed to (1) determine the mucosal global kinase activity and cytokine/chemokine profiles in IBD to increase our understanding of the inflammatory environment, (2) assess the possible association between baseline mucosal kinase activity or local cytokine/chemokine production with response to inhibition of JAK kinases with tofacitinib treatment, and (3) study changes in mucosal kinase activity during treatment with tofacitinib.

## 2. Material and methods

### 2.1. Study population

Between March 2018 and June 2020 participants were included in the Netherlands. For the IBD cohort and tofacitinib cohort 1, 48 participants were recruited from the outpatient clinic of the University Medical Center Utrecht and the Rijnstate Crohn and Colitis Centre, Arnhem. For tofacitinib cohort 2, 16 participants were enrolled at the Amsterdam University Medical Center.

#### 2.1.1. IBD cohort

**Figure 1.**
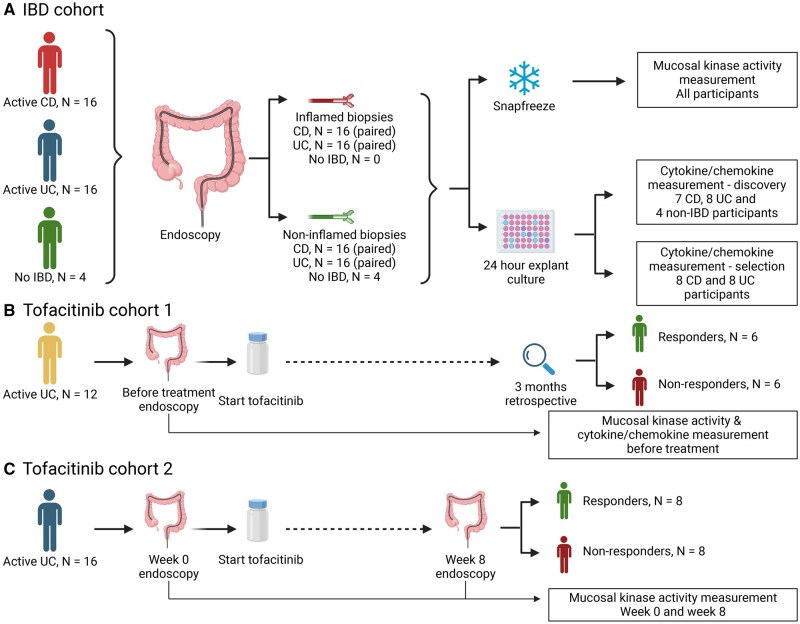
Study outline. The study contains three cohorts. (A) In the IBD cohort, 32 patients with active IBD (16 CD and 16 UC patients) and four non-IBD participants were enrolled. For the patients with CD and UC, both inflamed and non-inflamed colonic biopsies from the same patient were collected. For the non-IBD controls one colonic biopsy was collected for kinase activity analyses and five were collected for cytokine/chemokine analyses. Samples were either directly snap-frozen using liquid nitrogen or dry ice with subsequent storage at −80 °C and later used for kinase activity measurement, or were cultured for 24 hours for later cytokine/chemokine measurement. The cytokine/chemokine measurement was split-up in a discovery cohort in which a 65-analyte panel was used and a validation cohort in which a selection of these analytes (32-analyte panel) was used. (B) Tofacitinib cohort 1 included 12 UC patients with active disease in whom colonic mucosa biopsies were collected at baseline before start of tofacitinib treatment. Biopsies were snap-frozen using liquid nitrogen or dry ice for later kinase activity assessment or were directly cultured for later cytokine/chemokine assessment. Response to tofacitinib was retrospectively assessed, based on clinical and biochemical parameters. Two samples, both from responders, were excluded for the cytokine/chemokine analyses, because one was cultured for too long and one measurement did not pass quality control. During the STK measurement of one sample from a responder a technical error occurred, leaving five responders and six non-responders in the STK analyses. (C) Tofacitinib cohort 2 consists of 16 UC patients with active disease. These patients underwent an endoscopy at baseline, during which biopsies of inflamed colonic mucosa were taken, and were subsequently treated with tofacitinib 10 mg BID. After 8 weeks a second endoscopy was performed to assess response and to take colonic mucosal biopsies at the same location as the inflamed biopsies were previously taken. Treatment response was defined as a Mayo endoscopic score of 0 or 1 at week 8. For this study eight responders and eight non-responders were included. From the snap-frozen biopsies at week 0 and week 8 the colonic mucosal kinase activity was assessed. CD, Crohn’s disease; IBD, inflammatory bowel disease; N, number of participants; STK, serine/threonine kinase, UC, ulcerative colitis.

Sixteen CD and 16 UC patients were enrolled in the IBD cohort (Figure 1A). Eligible patients were ≥16 years of age, biological naïve, and scheduled to undergo an ileo-colonoscopy for suspected disease activity. Only patients who did not use corticosteroids within 3 months before sample collection were included. Mucosal biopsies were taken from optically inflamed and non-inflamed mucosa of the colon in both UC and CD patients. Colonic mucosal biopsies from non-IBD individuals (*n* = 4) served as controls.

#### 2.1.2. Tofacitinib cohort 1

UC patients (*n* = 12, ≥18 years of age), initiating tofacitinib (10 mg *bis in die* [BID] for 8-16 weeks, followed by 5 mg BID) were included in tofacitinib cohort 1 (Figure 1B). Before start of treatment biopsies were taken from the inflamed colonic mucosa. The response to tofacitinib at 3 months was retrospectively assessed, based on clinical and biochemical parameters. For example, patients were classified as non-responders if tofacitinib was stopped before 3 months because of therapy failure, if a patient underwent a colectomy within 3 months after start of tofacitinib, or if fecal calprotectin levels (if available) were above 250 µg/g. For all patients response classification was finally based on consensus between two authors (ECB and BO).

#### 2.1.3. Tofacitinib cohort 2

Sixteen UC patients, eight responders and eight non-responders, were selected from a larger prospective real-world cohort of patients initiating tofacitinib induction therapy^8^ (tofacitinib cohort 2, Figure 1C). Eligible patients were aged ≥18 years, had been diagnosed with UC ≥4 months before baseline, and had previously failed oral corticosteroids, thiopurines, or anti-TNFα therapy. Patients had to have active disease defined as a total Mayo score ≥6 and an endoscopic subscore ≥2 (ranging from 0 to 3, with higher scores indicating more severe inflammation). Patients were treated with tofacitinib 10 mg BID for 8 weeks. Colonic mucosal biopsies were taken from endoscopically inflamed mucosa at baseline, and from the same area 8 weeks after initiation of tofacitinib. At both timepoints the Mayo endoscopic score was centrally read by two blinded endoscopists. Response was defined as a Mayo score of 0 or 1 at week 8.

### 2.2. Sample processing

#### 2.2.1. Biopsies for kinase activity analyses

Biopsies were snap-frozen in liquid nitrogen or on dry ice and stored at −70 to −80 °C until further processing for kinase activity analyses.

#### 2.2.2. Explant culture for multiplex protein analyses

Biopsies were collected in Hank’s basic salt solution (HBSS) medium without calcium and magnesium (Gibco), containing 2% filtered fetal calf serum (Thermo Fisher Scientific, Life Technologies), 1% penicillin/streptomycin (Gibco), and 0.2% amphotericin B (Gibco) on ice. Biopsies were weighed to determine their mass, and subsequently cultured in 400 µL Roswell Park Memorial Institute (RPMI) medium (Gibco) supplemented with 10% filtered fetal calf serum, 1% penicillin/streptomycin, 1% l-glutamine (Thermo Fisher Scientific, Life Technologies), and 0.2% amphotericin B in a 48-well plate for 24 h at 37 °C and 5% CO_2_. Duration of culture was chosen to balance time-to-effect and viability of the biopsy. After culturing, the supernatant was spun down at 1600 rpm for 4 min at room temperature and stored at −70 to −80 °C until further processing for multiplex protein analyses.

### 2.3. Kinase activity

Kinase activity was determined employing the PamGene platform (PamGene International).^9^ This platform is based on measurement of phosphorylation of 13 amino acid–long peptide sequences containing tyrosine or serine/threonine phosphosites. These peptides are immobilized on a specific location on a porous aluminum oxide microarray. Four identical arrays on each PamChip (PamGene International) contain either 195 peptides for protein tyrosine kinase (PTK) or 142 peptides for serine/threonine kinase (STK) activity measurement. Twelve biopsy lysates, one per array, were measured simultaneously on three chips. The lysates were pumped through the array in the presence of ATP, resulting in phosphorylation of peptides by protein kinases in the lysates. FITC-labeled antibodies were used to detect the phosphorylation, captured by a 12-bit charge-coupled device (CCD) camera at different exposure times generating real-time kinetics data.

#### 2.3.1. Measurement

Biopsies that had been stored at −70 to −80 °C were transferred to a round-bottom Eppendorf, containing a steel bead and 120 µL lysis buffer consisting of mammalian protein extraction reagent (M-PER, Thermo Fisher Scientific) with 1% Halt protease inhibitor cocktail EDTA-free (Thermo Fisher Scientific) and 1% Halt phosphatase inhibitor cocktail (Thermo Fisher Scientific). The biopsies were then lysed in an oscillator for 2 min at a frequency of 50/s, following by 30 min of incubation on ice. Afterwards the lysates were spun down for 15 min at 4 °C at 16 000 *g*, aliquoted and put on dry ice, and subsequently stored at −80 °C until further use. Protein concentrations were determined with the Pierce bicinchoninic acid (BCA) protein assay kit (Thermo Fisher Scientific) according to the manufacturer’s protocol using the CLARIOstar (BMG Labtech).

Kinase activity was measured on the PamStation 12 according to the manufacturer’s specifications. For both PTK and STK measurement the microarrays were first blocked with 30 µL 2% bovine serum albumin (BSA) in a 30-min-long cycle to prevent non-specific binding. After blocking, the arrays were washed with 1× protein kinase buffer (PK buffer, PamGene International).

For PTK measurement, 5 µg lysate protein in combination with 1× PK buffer, 400 µM ATP (PamGene International), 0.01% BSA, fluorescein isothiocyanate-labeled antiphosphotyrosine antibody (FITC-PY20, PamGene International), 10 mM dl-dithiothreitol (DTT, PamGene international), 1× PTK additive (PamGene International), and Milli Q water in a total volume of 40 µL were loaded on the chips. The sample mix was then pumped up and down for 60 min through the porous membrane, with fluorescence being captured in real-time by the CCD camera.

For STK measurement, 1 µg lysate protein in combination with 1× PK buffer, 400 µM ATP, 0.01% BSA, STK primary antibody mix (PamGene International), and Mili Q water in a total volume of 40 µL were loaded on the chips. The sample mix was then pumped up and down for 60 min through the porous membrane. Next a washing step with 1× PBS and 0.01% Tween was performed, followed by loading of 30 µL detection mix containing 1× antibody buffer (PamGene International), FITC-labeled STK-antibody (PamGene International), and Milli Q water. The STK detection mix was pumped up and down for 30 min through the porous membrane, with fluorescence being captured by the CCD camera.

#### 2.3.2. Analyses

Signal intensities were quantified by automated image analyses in BioNavigator v.6.3 (PamGene International) taking local background and exposure time into account. Further analyses were performed in BioNavigator interfaced to R v.3.3.3. Quality control was performed, based on the overall strength of the signals (for PTK-based phosphorylation, at least 25% of the time increase in signal strength at a phosphor-site, and variance of <50% measured over all samples for STK-based phospho-sites; Table S1). Subsequently, data were log-transformed and ComBat batch correction^10^^,^^11^ (based on a Bayesian approach) was applied to correct for potential batch effects between different chips and runs.

Differences in peptide phosphorylation were tested using t-tests and displayed as volcano plots. The peptide phosphorylation data were utilized to predict the putative upstream kinase activity using the upstream kinase activity (UKA) tool (PamGene International). To this end, information from six different databases on *in vivo*/*in vitro* proven and *in silico* predicted potential phosphorylation of peptides by kinases was used. The iterative prediction algorithm, taking certainty of the possible phosphorylation and the number of peptides into account, calculates the kinase statistic (showing the direction and magnitude of the effect), the specificity score (a permutation based score on how likely this prediction could also be based on a random set of other peptides, higher scores indicating more specificity), and the significance score (a permutation-based score comparing two conditions). The latter two are then combined into a final kinase score. Since iterative predictions are performed per kinase, these measures are reported as medians or means. A cut-off for the median final kinase score >1.3 was used to determine the relevant predicted active kinases. To prevent spurious predictions, we set the minimum number of peptides needed to predict upstream kinase activity at three peptides. This led, for example, to no predictions about TYK2 activity. Results are displayed on human kinome trees^12^ showing the kinase family structure, and in tables.

### 2.4. Multiplex protein assay

#### 2.4.1. Measurement

Concentrations of 65 (cytokine/chemokine measurement discovery part of the IBD cohort) and 32 soluble factors (cytokine/chemokine measurement selection part of the IBD cohort & tofacitinib cohort 1) (Table S2) were measured simultaneously per panel in the 24-h explant biopsy culture supernatants by an in-house immunoassay based on Luminex technology (xMap, Luminex, Austin, TX, USA) at the Multiplex Core Facility (University Medical Center Utrecht) as previously described.^13^ The composition of the discovery panel was based on known associations with IBD and inflammation in general. Based on the findings in the discovery phase, a sub-selection was made for the cytokine/chemokine measurement in the selection part of the IBD cohort and tofacitinib cohort 1.

#### 2.4.2. Analyses

To ensure the quality of the data, we excluded samples for which in ≥30% of analytes <20 beads were measured, and we excluded analytes for which in ≥30% of samples <20 beads were measured. In addition, analytes were excluded if ≥30% of measurements were above the upper or below the lower limit of detection. Original (extrapolated) values were kept for traceable analytes below or above the limit of detection, and values that could not be extrapolated were replaced with 0.5 times the minimum value or 2 times the maximum value respectively. All analyte concentrations were divided by the biopsy mass to correct for biopsy mass.

We analyzed profiles of locally produced cytokines and chemokines by principal component analyses (PCAs) based on mean-centered and scaled values per analyte showing principal components 1 and 2, and centroids (ie, the mean of the coordinates per group). To assess whether cytokine/chemokine profiles differ per condition (ie, inflamed, non-inflamed, CD, UC, and no IBD) we performed pairwise permutational multivariate analysis of variance (PERMANOVA) based on Euclidean distance matrices of log-normalized cytokine/chemokine levels. Furthermore, we analyzed the cytokine/chemokine profiles using volcano plots (based on log_2_ fold change) and radar plots (showing the Box–Cox transformed mean values). All analyses were performed in R v.4.2.3 for macOS.^14^ PERMANOVA and two-sided t-test *P*-values were corrected for multiple testing by the Benjamini–Hochberg method. A false discovery rate (FDR) <0.05 was considered statistically significant.

### 2.5. Baseline characteristics

Baseline characteristics are displayed as numbers and proportions for categorical variables, and as medians with the 25^th^ and 75^th^ percentile (p25 and p75) for continuous variables.

### 2.6. Ethical considerations

The study protocols (TCBio 17/443 and 17/444, and NL62103.091.17 and NL57944.018.16) were approved by the Biobank Committee of the University Medical Center Utrecht (Utrecht, The Netherlands), the Research Ethics Committee of the Radboud University Medical Centre Nijmegen (CMO Region Arnhem-Nijmegen, Nijmegen, The Netherlands), and the Research Ethics Committee of the Amsterdam Academic Medical Center (Amsterdam, the Netherlands) respectively. All participants provided informed consent. The procedures were performed in accordance with the *Declaration of Helsinki*. All authors had access to the study data and had reviewed and approved the final manuscript.

### 2.7. Data availability statement

The data underlying this article will be shared on reasonable request to the corresponding author.

## 3. Results

### 3.1. Kinase activity and mucosal cytokine and chemokine profiles in IBD

#### 3.1.1. Baseline and sample characteristics

We included colonic mucosal biopsies of 16 patients with UC, 16 with CD, and four non-IBD controls in the IBD cohort (55.6% females, median age 42.5 years, Figure 1A). The median endoscopic Mayo score was 2 for the UC patients, and the median simple endoscopic score for CD patients (SES-CD) was 11.5 (Table 1). Most IBD patients were not receiving IBD medication (53%), and 44% of patients used 5-aminosalicylic acid compounds.

**Table 1.** Baseline and sample characteristics.

	IBD cohort			Tofacitinib cohort 1	Tofacitinib cohort 2
	Kinase activity analyses	Cytokine analyses Discovery (all analytes)^a^ ^,^ ^b^	Cytokine analyses Selection of analytes^b^	Kinase activity and cytokine analyses^c^	Kinase activity analyses
	*N* = 36	*N* = 19	*N* = 16	*N* = 12	*N* = 16
	*n* = 68	*n* = 48^a^	*n* = 32	*n* = 12	*n* = 32
***Demographics and clinical characteristics***					
**Female sex**	20 (55.6%)	10 (52.6%)	9 (56.3%)	7 (58.3%)	13 (81.3%)
**Age (years)**	42.5 (31.8, 51.8)	47 (38, 58)	33 (30.5, 43.5)	39 (35.5, 44.5)	43 (26.8, 51.8)
**Current smoker**	5 (13.9%)	2 (10.5%)	2 (12.5%)	3 (25%)	1 (6.3%)
** Missing:**	—	—	—	1	—
***IBD characteristics***					
**IBD phenotype**					
** Ulcerative colitis**	16 (44.4%)	8 (42.1%)	8 (50%)	12 (100%)	16 (100%)
** Crohn’s disease**	16 (44.4%)	7 (36.8%)	8 (50%)	—	—
** No IBD**	4 (11.1%)	4 (21.1%)	—	—	—
**IBD duration (years)^d^ ^,^ ^e^**	4.5 (0, 14.2)	9.3 (2.0, 16.7)	1.4 (0, 12.1)	7.6 (3.1, 13.3)	6 (4, 9.3)
**Location UC (Montreal classification)^f^**					
** E1 (ulcerative proctitis)**	7 (43.8%)	4 (50%)	3 (37.5%)	3 (25%)	0
** E2 (left-sided UC)**	4 (25.0%)	2 (25%)	2 (25%)	2 (16.7%)	6 (37.5%)
** E3 (extensive UC)**	5 (31.3%)	2 (25%)	3 (37.5%)	7 (58.3%)	10 (62.5%)
**Age of diagnosis CD (Montreal classification)^f^**					
** A1 (≤16 years)**	0	0	0	—	—
** A2 (17-39 years)**	15 (93.8%)	6 (85.7%)	8 (100%)	—	—
** A3 (≥40 years)**	1 (6.3%)	1 (14.3%)	0	—	—
**Location CD (Montreal classification)^f^**					
** L1 (ileum only)**	0	0	0	—	—
** L2 (colon only)**	5 (31.3%)	1 (14.3%)	4 (50%)	—	—
** L3 (ileocolonic)**	11 (68.8%)	6 (85.7%)	4 (50%)	—	—
**Behavior CD (Montreal classification)^f^**					
** B1 (non-stricturing, non-penetrating)**	11 (68.8%)	5 (71.4%)	5 (62.5%)	—	—
** B2 (stricturing)**	3 (18.8%)	1 (14.3%)	2 (25%)	—	—
** B3 (penetrating)**	2 (12.5%)	1 (14.3%)	1 (12.5%)	—	—
**Symptom-based activity score^d^ ^,^ ^g^**					
** UC: Short clinical colitis activity index—baseline**	7 (4.5, 8)	5 (4, 6.5)	8 (6.8, 8.3)	NA	11 (7, 13)
** UC: Short clinical colitis activity—week 8**	—	—	—	—	2 (1, 4.8)
** Missing**	1	1	0	—	0
** CD: Harvey–Bradshaw index**	8 (6.3, 10.8)	9 (5, 11)	7.5 (6.8, 11.5)	—	—
** Missing**	2	2	0		
**Fecal calprotectin level (µg/g)^d^ ^,^ ^h^**					
** Baseline**	201 (105, 540)	155 (97.8, 368)	422 (128, 592)	1620 (831, 1960)	3180 (1480, 6000)
** Days between measurement and biopsy**	13.5 (5.3, 23.5)	14 (5.3, 39.3)	12 (5.5, 20.5)	18 (4, 27.3)	7 (5, 12)
** Missing**	2	1	1	0	3
** Week 8**	—	—	—	—	380 (64.8, 1710)
** Serum CRP level (mg/L)^d^ ^,^ ^h^**	—	—	—	—	0 (0, 0)
** Baseline**	9.8 (0, 30.5)	9.5 (1.9, 26.5)	7 (0, 23)	10 (2.2, 18)	5.3 (1.5, 28.8)
** Days between measurement and biopsy**	14.5 (5.3, 42.5)	15 (6.5, 61)	11.5 (2, 19.8)	0 (0, 13.5)	5.5 (0, 10)
** Missing**	2	0	2	1	0
** Week 8**	—	—	—	—	1.9 (0.6, 6.2)
** Days between measurement and biopsy**	—	—	—	—	0 (0, 0)
**Endoscopic score^i^**					
** UC: Mayo endoscopic score—baseline**	2 (2, 2)	2 (2, 2.3)	2 (2, 2)	2 (2, 2.3)	3 (2.8, 3)
** UC: Mayo endoscopic score—week 8**	—	—	—	—	1.5 (1, 3)
** CD: SES-CD**	11.5 (9.3, 17)	17 (11, 17)	10.5 (6.8, 12.3)	—	—
** Missing**	2	2	0	—	—
**Current IBD-medication use^d^ ^,^ ^j^**					
** No IBD-medication—baseline**	17 (53.1%)	6 (40%)	10 (62.5%)	1 (8.3%)	3 (18.8%)
** No IBD-medication—week 8**	—	—	—	—	0
** Corticosteroids**	0	0	0	6 (50%)	10 (62.5%)
** 5-Aminosalicylic acid (5-ASA)**	14 (43.8%)	9 (60%)	5 (31.3%)	5 (41.7%)	7 (43.8%)
** Thiopurine**	2 (6.3%)	2 (13.3%)	0	4 (33.3%)	0
** Methotrexate**	1 (3.1%)	0	1 (6.3%)	1 (8.3%)	0
** Anti-TNF-α**	0	0	0	4 (33.3%)	0
** Vedolizumab (anti-integrin α4β7)**	0	0	0	3 (25%)	0
** Tofacitinib—baseline**	0	0	0	0	0
** Tofacitinib—week 8**	—	—	—	—	16 (100%)
**Response to tofacitinib *in vivo* ^k^**					
** Responders**	—	—	—	6 (50%)	8 (50%)
** Non-responders**	—	—	—	6 (50%)	8 (50%)
***Sample characteristics***					
**Hospital**					
** Hospital 1**	29 (80.6%)	12 (63.2%)	16 (100%)	1 (8.3%)	—
** Hospital 2**	7 (19.4%)	7 (36.8%)	0	11 (91.7%)	—
** Hospital 3**	—	—	—	—	16 (100%)
**Location of inflamed biopsies^d^ ^,^ ^l^**					
** Ascending colon**	6 (18.8%)	5 (33.3%)	0	1 (8.3%)	0
** Transverse colon**	6 (18.8%)	0	6 (37.5%)	0	0
** Descending colon**	3 (9.4%)	1 (6.7%)	2 (12.5%)	0	1 (6.3%)
** Sigmoid**	9 (28.1%)	4 (26.7%)	5 (31.3%)	2 (16.7%)	12 (75%)
** Rectum**	8 (25.0%)	5 (33.3%)	3 (18.8%)	9 (75%)	3 (18.8%)
**Location of non-inflamed biopsies^m^**					
** Ascending colon**	8 (22.2%)	5 (33.3%)	2 (12.5%)	—	—
** Transverse colon**	4 (11.1%)	0 (0%)	4 (25%)	—	—
** Descending colon**	6 (16.7%)	3 (20%)	3 (18.8%)	—	—
** Sigmoid**	14 (38.9%)	4 (26.7%)	6 (37.5%)	—	—
** Rectum**	4 (11.1%)	3 (20%)	1 (6.3%)	—	—

Abbreviations: CD, Crohn’s disease; CRP, C-reactive protein; IBD, inflammatory bowel disease; *N*, number of participants; *n*, number of biopsies; NA, not applicable; TNF, tumor necrosis factor, UC, ulcerative colitis.

Continuous variables are depicted as median (p25, p75), and categorical variables as number (proportion) unless indicated otherwise.

a Per non-IBD control five biopsies are included in the cytokine/chemokine analyses. Seven CD patients are included in this part, because one CD patient was included in the study after the moment the Luminex measurement was performed. Two samples (one inflamed UC and one non-inflamed UC sample from separate patients) were excluded from the analyses, because in ≥30% of analytes <20 beads were measured.

b A 65-analyte Luminex panel was used in the first part, and a 32-panel in the second part of the cytokine analyses (Table S2).

c For one patient the sample was cultured for too long and therefore no Luminex was performed. One other patient was excluded from the Luminex analyses, because for ≥30% of analytes <20 beads were measured. In total samples from 10 patients (four responders and six non-responders) were included in the Luminex analyses for this part. In the serine/threonine kinase analyses five responders and six non-responders are included due to a technical error while measuring the sample of one responder.

d IBD-related variables are only shown for IBD patients.

e In tofacitinib cohort 2 the duration of IBD is collected in complete years.

f All proportions for Montreal classification and endoscopy characteristics are only calculated for those participants to whom it applies.

g The clinical score was not available in tofacitinib cohort 1.

h If the measurement of calprotectin or CRP was below detection, we imputed 0.5 times the lower limit of detection.

i In tofacitinib cohort 2 the Mayo endoscopic score was centrally read at baseline and at week 8. For all other parts the endoscopic score was determined by the endoscopist during the endoscopy. In tofacitinib cohort 1 one patient has been included with a Mayo endoscopic score of 1; all other patients in tofacitinib cohort 1 had a Mayo endoscopic score of 2 or 3.

j For tofacitinib cohort 2 the medication use is the same at baseline and week 8, except for the use of tofacitinib. In tofacitinib cohort 2 patients who used 5-ASA or oral corticosteroids at baseline used these at least until week 8. The use of thiopurines or methotrexate within 2 weeks, and anti-tumor necrosis factor-α, vedolizumab, and ustekinumab within 8 weeks of baseline was prohibited in tofacitinib cohort 2.

k Response was defined in tofacitinib cohort 1 by retrospective assessment of clinical and biochemical parameters at 3 months. In four of six non-responders tofacitinib was stopped before 3 months, and two of these had a colectomy within 3 months after start of tofacitinib. Response was defined in tofacitinib cohort 2 as a centrally read Mayo endoscopic score of 0 or 1 at week 8.

l For tofacitinib cohort 2 the biopsies at baseline and at week 8 are taken at the same anatomic location in the colon. Therefore, the locations for the inflamed biopsies display the locations at baseline and at week 8.

m From the non-IBD controls five biopsies (from the five different locations within the colorectum) per patient were used in the cytokine/chemokine analyses; these are not reported in the table. For the kinase activity analyses biopsies from the descending colon were used, which are reported in the table.

#### 3.1.2. Kinase activity is broadly increased in IBD inflammation

**Figure 2.**
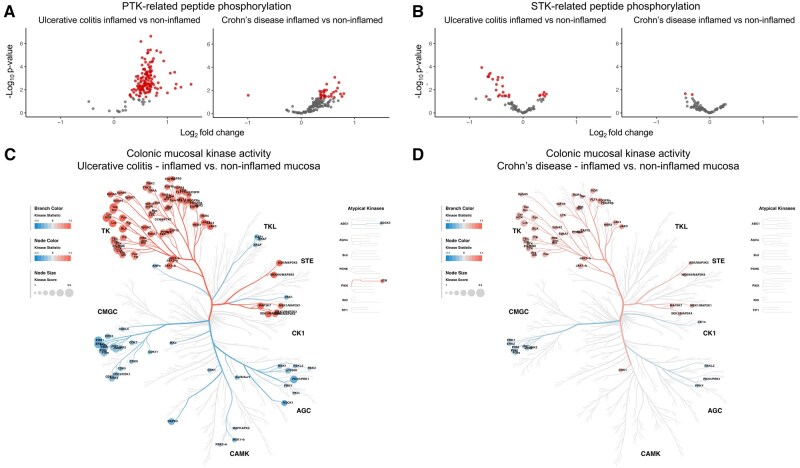
Colonic mucosal kinase activity profiles in ulcerative colitis and Crohn’s disease. (A–B) Volcano plots showing the log_2_ fold change in peptide phosphorylation comparing inflamed vs non-inflamed colonic mucosal biopsies for UC (*n* = 16) and CD (*n* = 16). Red circles depict peptides that are statistically significantly (*P *< .05) more (log_2_ fold change >0) or less (log_2_ fold change <0) phosphorylated in inflamed mucosa compared to non-inflamed mucosa. (A) Peptides measured in the protein tyrosine kinase (PTK) assay, and (B) measured in the serine/threonine kinase (STK) assay. (C–D) From these measurements, kinase activity is predicted. These predictions are based on (sets of) peptides that have *in vivo* or *in vitro* been shown to be phosphorylated by a specific kinase or that have *in silico* been shown to be (potentially) phosphorylated by a specific kinase, using an iterative process. To avoid spurious predictions, only kinases for which three or more peptides were present in the panel were included. Kinases with a median kinase score >1.3 were considered relevant and projected on the human kinome tree.^12^ The median kinase statistic (>0 indicates more kinase activity in inflamed mucosa, <0 indicates less kinase activity in inflamed mucosa compared to non-inflamed mucosa) determines the color of the circles per kinase; the size of the circles is proportionate to the median kinase score. (C) Kinase activity for UC, and (D) kinase activity for CD comparing inflamed to non-inflamed colonic mucosa. CD, Crohn’s disease; *n*, number of patients; PTK, protein tyrosine kinase; STK, serine/threonine kinase; UC, ulcerative colitis.

Biopsies from inflamed colonic mucosa from UC and CD patients exhibited statistically significant (*P *< .05) mostly increased peptide phosphorylation as compared to non-inflamed tissue in the PTK assays (UC: 135 peptides increased, CD: 30 peptides increased and one peptide decreased, Figure 2A and Table S3). Conversely, in the STK analyses, we observed both increased (six peptides) and decreased (21 peptides) peptide phosphorylation in inflamed mucosa of UC. In CD, only for three STK-related peptides was a significantly decreased phosphorylation observed (Figure 2B, Table S4). Analysis of predicted upstream kinase activity revealed a broad increase in predicted kinase activity in inflamed UC mucosa compared to non-inflamed mucosa, predominantly within the tyrosine kinase families, including the JAK family (Figure 2C, Table S5). In CD, a similar profile of activated kinases was observed when comparing inflamed to non-inflamed mucosa, although it was less pronounced (ie, lower kinase statistic, lower kinase score, and fewer kinases) (Figure 2D, Table S6). Direct comparison of inflamed colonic mucosa of UC and CD patients showed higher kinase activity, particularly within the tyrosine kinase families, including the JAK kinases, in inflamed colonic mucosa in UC. For non-inflamed mucosa mainly a few serine/threonine kinases were found to be more active in UC compared to CD (Figure S1). When comparing inflamed mucosa of UC patients to non-IBD controls (*n* = 4) an increase in activity of tyrosine kinases, but also serine/threonine kinases was noted, without any decreased kinase activity (Figure S2). Again, for CD an increase in kinase activity was also noted when comparing inflamed mucosa to non-inflamed mucosa of non-IBD controls, but to a lesser extent than in UC.

In conclusion, both in CD and UC inflammation is associated with distinct mucosal kinase activity profiles. These profiles are characterized by an increase in tyrosine kinase activity, which extends beyond JAK kinases, and a variable pattern of increased and decreased activity among serine/threonine kinases. The mucosal kinase activity, particularly within the tyrosine kinase family, was considerably higher in inflamed mucosa in UC compared to CD.

#### 3.1.3. Cytokine and chemokine profiles

To determine whether the differences found in kinase activity between inflamed and non-inflamed mucosa were also reflected in cytokine and chemokine profiles, we assessed spontaneous mucosal cytokine and chemokine production corrected for biopsy mass in 24-h non-stimulated explant cultures of colonic mucosa. First, we measured in a discovery cohort the concentration of 65 analytes of which 58 passed our quality control (Table S2), from eight UC and seven CD patients and from four non-IBD controls, all included in the previously mentioned kinase activity measurements. Two samples (one inflamed UC sample and one non-inflamed UC sample from different patients) did not pass quality control, and for one non-inflamed UC sample the biopsy mass was missing. These three samples were excluded from the analyses.

In PCA the mucosal cytokine and chemokine profile of inflamed tissue was clearly distinct from the profiles of non-inflamed and non-IBD control mucosa (Figure S3A and Table S7, PERMANOVA FDR <0.05 comparing inflamed samples to non-inflamed samples, Table S8). The loadings of the PCAs indicate that markers related to innate immune activation, such as C–C motif chemokine ligand-2 (CCL2), CCL3, CCL4, oncostatin M, granulocyte–macrophage colony-stimulating factor (GM-CSF), IL1β, S100A8, and IL10, are more oriented towards the centroid of the inflamed CD samples, suggesting that these markers are more strongly associated with CD inflammation. On the other hand, the loadings of markers related to adaptive immunity, including IL17, IL7, IL4, IL5, and IL21, are more oriented towards the centroid of inflamed UC samples (Figure S3A). PCAs based on the concentration of 32 cytokines and chemokines in the selection phase of the IBD cohort confirmed the distinct separation of inflamed and non-inflamed samples (PERMANOVA FDR < 0.05 comparing inflamed CD vs non-inflamed CD and inflamed UC vs non-inflamed UC, Table S9) (Figure S3B and Table S10). The log_2_ fold change in cytokine and chemokine concentrations in the discovery part of the IBD cohort revealed statistically significantly increases in 15 analytes in inflamed UC samples, and 20 analytes in inflamed CD samples of which nine analytes overlapped (FDR < 0.05, log_2_ fold change > 0.5, Figure S3C and D). Among these analytes were cytokines that are well-established in IBD such as S100A8 (which together with S100A9 forms the heterodimer calprotectin), as well as more recently implicated makers such as oncostatin M, elevated in both UC and CD and triggering receptor expressed on myeloid cells 1 (TREM1) in CD. Also, pro-inflammatory proteins, such as IL6, IL1β, GM-CSF, and CCL3 were increased in both UC and CD. We found an overall comparable spontaneous produced mucosal cytokine and chemokine profile in UC and CD, despite the observed differences in the magnitude and profiles of kinase activity at the mucosal level between UC and CD.

### 3.2. Kinase activity and cytokine/chemokine profiles in association with tofacitinib treatment

#### 3.2.1. Baseline and sample characteristics of tofacitinib cohorts 1 and 2

We assessed the baseline (before treatment) mucosal kinase activity, and cytokine and chemokine profiles in colonic biopsies of 12 UC patients (six responders vs six non-responders for the PTK analyses, and due to a technical error five responders vs six non-responders for the STK analyses) initiating tofacitinib in tofacitinib cohort 1 and the baseline mucosal kinase activity in 16 UC patient (eight responders, eight non-responders) in tofacitinib cohort 2 (Figure 1B–C). The proportion of female patients was lower in tofacitinib cohort 1 compared to tofacitinib cohort 2 (58.3% vs 81.3%) (Table 1). At baseline, patients in tofacitinib cohort 1 had lower median fecal calprotectin levels (1620 vs 3180 µg/g), but higher median C-reactive protein (CRP) levels (10 vs 5.3 mg/L), compared to patients in tofacitinib cohort 2. The use of corticosteroids and 5-aminosalicylic acid was comparable in both cohorts, but patients who were recently using thiopurines, methotrexate, anti-TNFα agents, or vedolizumab were excluded in tofacitinib cohort 2, while up to 33.3% of patients had used any of these medications in tofacitinib cohort 1. Clinical, biochemical (ie, CRP and calprotectin levels), and endoscopic score data in responders and non-responders per tofacitinib cohort can be found in Table S11.

#### 3.2.2. Association of baseline mucosal kinase activity and cytokine/chemokine profiles with response to tofacitinib

**Figure 3.**
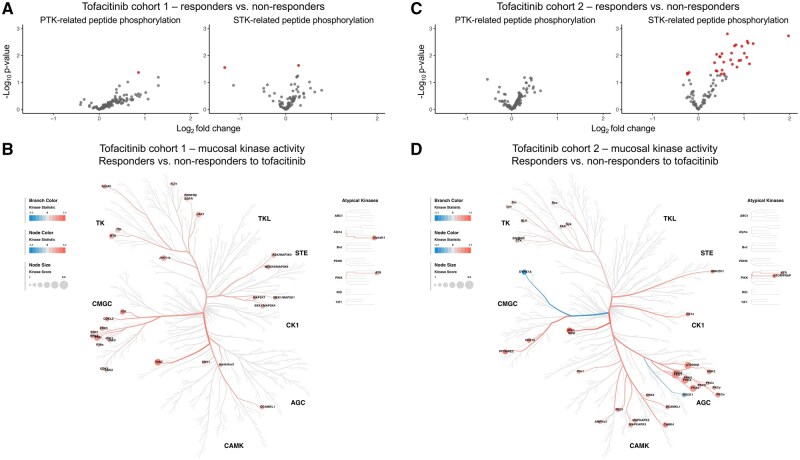
Colonic mucosal kinase activity profiles in ulcerative colitis patients before the start of tofacitinib comparing responders to non-responders. (A&C) Volcano plots showing the log_2_ fold change in peptide phosphorylation comparing responders to non-responders to tofacitinib. Red circles depict peptides that are statistically significantly (*P *< .05) more (log_2_ fold change >0) or less (log_2_ fold change <0) phosphorylated in the mucosa of responders compared to non-responders. (A) Phosphorylation per peptide in tofacitinib cohort 1 (six responders vs six non-responders for the PTK analyses, due to a technical error five responders vs six non-responders were measured in the STK analyses). (C) Phosphorylation per peptide in tofacitinib cohort 2 (eight responders vs eight non-responders). (B&D) Human kinome trees^12^ displaying relevant kinase activity (predicted kinases with a median kinase score >1.3 are displayed) comparing responders to non-responders (kinase statistic >0 depicts kinases that are more active, and <0 depicts kinases that are less active in responders compared to non-responders). (B) The kinase activity profile in tofacitinib cohort 1, and (D) the kinase activity profile in tofacitinib cohort 2 of this study. Although overall mucosal kinase activity is increased in responders compared to non-responders, only two predicted kinases (ie, ATR and DCLK1 [also known as DCAMKL1]) overlap between the two cohorts (Table S3). ATR, ataxia telangiectasia and Rad3-related protein; DCLK1, doublecortin like kinase 1; PTK, protein tyrosine kinase; STK, serine/threonine kinase.

Baseline phosphorylation of one PTK- and one STK-related peptide was significantly (*P *< .05) increased, and phosphorylation of one STK-related peptide was decreased in responders compared to non-responders in tofacitinib cohort 1 (Figure 3A, Tables S12 and S13). Twenty-nine upstream kinases were predicted to be more active in responders compared to non-responders (Figure 3B, Tables S14 and S15). In the independent cohort of 16 UC patients (tofacitinib cohort 2), the phosphorylation of 28 STK-related peptides was increased and decreased for three STK-related peptides in responders compared to non-responders (Figure 3C, Tables S16 and S17). The kinase activity of 35 upstream kinases was predicted to be more active, and two to be less active in responders compared to non-responders (Figure 3D, Tables S14 and S18). Only two predicted kinases overlapped between the cohorts: doublecortine-like kinase 1 (DCLK1, also known as DCAMKL1) and ataxia telangiectasia and Rad3-related protein (ATR). Spontaneously produced mucosal cytokines and chemokines at baseline were not found to be associated with response to tofacitinib (Figure S4 and Table S19). Thus, here we demonstrate that increased mucosal kinase activity before the start of tofacitinib is associated with response to tofacitinib in two separate cohorts of whom only two predicted kinases overlapped between the cohorts, however.

#### 3.2.3. Kinase activity change during treatment with tofacitinib

**Figure 4.**
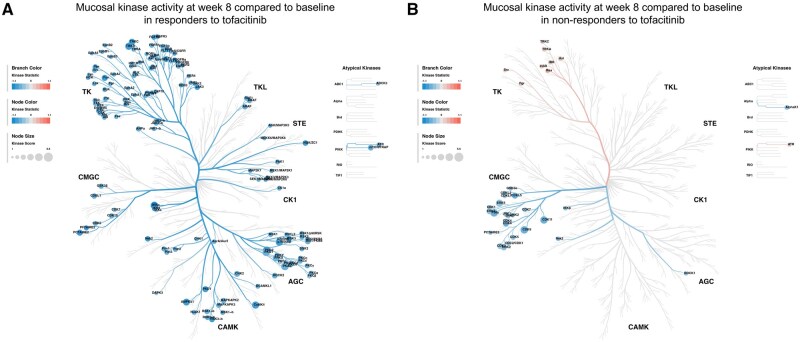
Colonic mucosal kinase activity profiles in ulcerative colitis patients after 8 weeks of tofacitinib treatment compared to before start of treatment. (A–B) Predicted kinase activity is plotted on the human kinome tree.^12^ A kinase statistic <0 represents kinases that are less active after 8 weeks of tofacitinib treatment compared to baseline values in tofacitinib cohort 2. Only kinases with a median kinase score >1.3 are displayed. (A) Results for the eight UC patients who responded to tofacitinib, and (B) the kinase activity profile for the eight UC patients who did not respond to tofacitinib.

Next, we assessed the changes in mucosal kinase activity profiles in UC patients treated with tofacitinib (tofacitinib cohort 2). Therefore, we compared the mucosal kinase activity after 8 weeks of treatment with tofacitinib to baseline kinase activity in responders (*n* = 8) and non-responders (*n* = 8). In responders, treatment with tofacitinib for 8 weeks was associated with decreased activity not only in JAK kinases, but also in a broad spectrum of PTKs and STKs (Figure 4A, Table S20). Nearly all kinases (34 out of 35) that showed increased baseline activity in responders versus non-responders exhibited decreased activity after 8 weeks of tofacitinib treatment in responders. In non-responders, 8 weeks of tofacitinib treatment was associated with decreased activity primarily in extracellular signal regulated kinase (ERK-), cyclin-dependent kinase (CDK-), and c-Jun N-terminal kinase (JNK)-family activity, while JAK kinase activity remained unchanged (Figure 4B, Table S21). Comparison of responders to non-responders after 8 weeks of treatment with tofacitinib demonstrated a lower tyrosine kinase activity in responders, including JAK2 and JAK3 (Figure S5). Five out of 35 kinases (ie, ATR, Src, focal adhesion kinase 1 [FAK1], cytoplasmic tyrosine kinase [CTK also known as MATK], and adenosine monophosphate-activated protein kinase-α1 [AMPKα1]) for which increased activity at baseline was associated with treatment response were decreased in kinase activity in responders compared to non-responders after 8 weeks of tofacitinib treatment.

These findings not only reflect a change in inflammation status in responders (transitioning from inflamed to non-inflamed), but also demonstrate a broad impact of (pan)JAK inhibition on mucosal kinase activity. Furthermore, the majority of kinases with increased activity at baseline in responders compared to non-responders show a decrease in activity in responders after 8 weeks of tofacitinib treatment, highlighting their potential pre-treatment association with tofacitinib response.

## 4. Discussion

This study demonstrates that mucosal kinase activity profiles are closely associated with active inflammation in IBD, with notable increases in tyrosine kinase activity, and decreased activity in some serine/threonine kinase families. Overall, the magnitude of change in kinase activity was higher in the mucosa of UC patients than in CD patients, underscoring potential differences in disease pathophysiology. On the other hand, cytokine/chemokine profiles related to inflamed mucosa largely overlapped between UC and CD in a non-stimulated 24-h explant culture assay. Response to tofacitinib in UC patients was associated with increased mucosal kinase activity at baseline, although only two predicted kinases (ie, ATR and DCLK1) were consistently identified in two independent cohorts. A strong reduction in overall mucosal kinase activity was observed in responders, but not in non-responders to tofacitinib.

Interestingly, the amplitude and signature of the kinase activity in the inflamed colonic mucosa differed considerably between UC and CD patients, highlighting the presence of differences in pathophysiology. We observed increased activity of JAK1, JAK2, and JAK3 (TYK2 was not included in the analyses) in the inflamed mucosa of patients with UC compared to both non-inflamed UC mucosa and inflamed mucosa from patients with CD. Additionally, JAK1 and JAK3 exhibited greater activity in inflamed CD mucosa compared to non-inflamed CD mucosa and in inflamed UC mucosa compared to non-inflamed non-IBD mucosa. Notably, only JAK3 from the JAK family showed increased activity in inflamed CD mucosa compared to non-inflamed non-IBD mucosa. These differential patterns of JAK family kinase activity may partly explain why tofacitinib and filgotinib are approved for UC but not for CD. However, the efficacy of upadacitinib for both diseases suggests that other mechanisms also contribute. Of note, in addition to differences in the activity of JAK-family kinases, other kinase families were found to be more active in UC inflammation, such as the fibroblast growth factor receptor (FGFR) and the ephrin (Eph) family. Increased activity for these kinase families is in line with previous data from a limited kinase phosphorylation screen of colonic tissue in IBD.^15^ FGFR signaling plays a role in multiple processes, including intestinal development, homeostasis, tissue repair,^16^ and macrophage polarization.^17^ Eph signaling is among others involved in vascular permeability, leukocyte trafficking, and immune cell activation.^18^^,^^19^ The exact role and importance in IBD-related inflammation of these kinase families still needs to be elucidated. Overall, our data reveal that UC and CD have distinct mucosal kinase activity profiles, providing further mechanistical support for their classification as discrete clinical entities. Our results may therefore serve as a foundation for future targeted investigations into the role of kinase activity in the pathogenesis and treatment of IBD.

The local spontaneous production of a broad spectrum of pro-inflammatory cytokines/chemokines including IL1β, IL6, S100A8 (a part of calprotectin), GM-CSF, chemokine (C–X–C motif) ligand 13 (CXCL13), CCL3, matrix metalloproteinase 9 (MMP9), and MMP10 in both UC and CD reflects the heightened activity of both immune and non-immune cells. Furthermore, oncostatin M and TREM1, both of which have been associated with the response to anti-TNFα therapy,^20^^,^^21^ were produced in high quantities in inflamed mucosa of UC (oncostatin M) and CD (oncostatin M and TREM1) patients in our explant-culture model.

There is currently a high unmet need to establish predictive markers of treatment response in IBD, as this could help to tailor therapeutic strategies, and avoid a cumbersome treat-and-evaluate process.^22^ Different approaches have already been explored, with varying results, of which none of the reported predictors have been implemented in clinical practice. Two studies have identified associations between the partial Mayo score and CRP levels and response to tofacitinib.^23^^,^^24^ Joustra et al. identified a mucosal epigenetic signature correlated with treatment response to tofacitinib,^25^ and more recently a tofacitinib-organoid model and/or staining for colonic multidrug and toxin extrusion protein 1 (MATE1) expression has been suggested to be correlated to tofacitinib response.^26^ While mucosal epigenetic signatures and other novel approaches have shown promise, their integration into clinical practice depends on robust external validation. As an example, the randomized PROFILE trial underscored the challenges in translating predictive tools into practice by demonstrating that a promising 17-gene blood-based prognostic profile did not show clinical utility in the prediction of anti-TNFα response nor selection of patients who would need early advanced therapy, despite previous rigorous evaluation.^27^

For prediction studies, it is always a question of whether to focus on broad patterns or specific elements; this raises the question of whether pursuing kinome-wide screening or a kinase-focused approach is a better strategy to identify predictive markers of treatment response in patients with IBD. Our study observed in two independent cohorts that responders to tofacitinib exhibited higher overall kinase activity in the colonic mucosa before treatment. However, only for two specific kinases (ie, ATR and DCLK1) was increased activity observed in both cohorts. ATR is a serine/threonine kinase that can recognize DNA damage and thereby plays a role in the DNA damage checkpoint and can hold cell cycle progression.^28^ ATR activation has previously been found to be an effect of 5-aminosalicylic acid in colorectal cancer cell lines.^29^ DCLK1 (also known as DCAMKL1) is mainly expressed in tuft cells,^30^ and has been linked to inflammatory responses to pathogens,^31^^,^^32^ but has also been suggested to ameliorate chronic colitis in a mouse model.^32^ Both kinases have, to our knowledge, not previously been linked to treatment response in IBD. It is, however, important to emphasize that our results are based on predicted upstream kinase activity, and it was not the aim of our study to single out sole kinases. The variability in predicted kinase activity profiles associated with response to tofacitinib may be explained by the relatively small sample size, different response definitions, differences in pre-treatment conditions (eg, use of concomitant IBD medication), disease heterogeneity, or the complexity of identifying a singular kinase associated with treatment response. Of note, in a single-cell RNA sequencing-based study, increased mucosal JAK–STAT activity was correlated with response to tofacitinib, while non-responders had increased NF-kB pathway activity pre-treatment.^33^ This is thus partly in line with our observation of increased kinase activity in responders, although we did not find an increase in kinase activity in non-responders. Finally, our observation that treatment response to tofacitinib correlates with a general reduction in kinase activity at the level of the mucosa suggests that (successful) treatment with tofacitinib ultimately leads to a broader modulation of mucosal kinase signaling than only the targeted pathways.

Our study has several strengths. We studied both the mucosal kinase signature and the cytokine and chemokine profiles in patients not using corticosteroids or biologicals. This enabled a comprehensive exploration of the mucosal kinase activity and cytokine/chemokine landscape in the setting of CD and UC, and the comparison of inflamed and non-inflamed tissue within the same patients. Furthermore, to the best of our knowledge, we are the first to investigate local kinase activity changes, beyond JAK kinases, in response to treatment with a JAK inhibitor, which we performed in two separate cohorts. In this regard our study provides valuable insights into both the potential associations between baseline kinase activity and the response to tofacitinib, as well as the mucosal kinase activity alterations induced by this JAK inhibitor.

Our study also has some limitations. Although we conducted a broad-scale screening of kinase activity using a peptide phosphorylation assay and subsequent kinase activity prediction, not all kinases, for example TYK2, were covered by this approach. Additionally, we did not validate the predictions employing methods such as immunoblotting of phosphorylated kinases, as our study did not aim primarily to identify single kinases. Because we utilized complete mucosal tissue biopsies, our results may reflect differences in cell composition rather than alterations in kinase activity per cell type. Future studies should focus on the role of specific kinases and their activity in the treatment response to JAK inhibitors in the setting of IBD. Moreover, exploring targets beyond the JAK–STAT pathway could be beneficial in the pursuit of alternative therapeutic avenues for treating IBD.

In conclusion, we show that the kinase activity profile of the inflamed mucosa is different from non-inflamed tissue in UC and CD, with different profiles per disease subtype. An increase in kinase activity was found to be associated with response to tofacitinib, although only two specific kinases could be replicated. When endoscopic response is induced by tofacitinib, the general kinase activity of the mucosa seems to return to its level in the non-inflammatory state. These findings highlight the potential of kinase activity profiling in better understanding the pathophysiology of IBD, and the differences in treatment responses between UC and CD and individual patients.

## Supplementary Material

jjaf174_Supplementary_Data

## References

[1] Dolinger M , Torres J, Vermeire S. Crohn’s disease. Lancet. 2024;403:1177-1191. 10.1016/S0140-6736(23)02586-2 38437854

[2] Le Berre C , Honap S, Peyrin-Biroulet L. Ulcerative colitis. Lancet. 2023;402:571-584. 10.1016/S0140-6736(23)00966-2 37573077

[3] Lasa JS , Olivera PA, Danese S, Peyrin-Biroulet L. Efficacy and safety of biologics and small molecule drugs for patients with moderate-to-severe ulcerative colitis: a systematic review and network meta-analysis. Lancet Gastroenterol Hepatol. 2022;7:161-170. 10.1016/S2468-1253(21)00377-0 34856198

[4] Barberio B , Gracie DJ, Black CJ, Ford AC. Efficacy of biological therapies and small molecules in induction and maintenance of remission in luminal Crohn’s disease: systematic review and network meta-analysis. Gut. 2023;72:264-274. 10.1136/GUTJNL-2022-328052 35907636

[5] Raine T , Danese S. Breaking through the therapeutic ceiling: what will it take? Gastroenterology. 2022;162:1507-1511. 10.1053/J.GASTRO.2021.09.078 34995533

[6] Salas A , Hernandez-Rocha C, Duijvestein M, et al JAK-STAT pathway targeting for the treatment of inflammatory bowel disease. Nat Rev Gastroenterol Hepatol. 2020;17:323-337. 10.1038/S41575-020-0273-0 32203403

[7] Seif F , Khoshmirsafa M, Aazami H, Mohsenzadegan M, Sedighi G, Bahar M. The role of JAK-STAT signaling pathway and its regulators in the fate of T helper cells. Cell Commun Signal. 2017;15:23. 10.1186/S12964-017-0177-Y 28637459 PMC5480189

[8] van Gennep S , Fung ICN, Jong DCd, et al Histological outcomes and JAK-STAT signalling in ulcerative colitis patients treated with tofacitinib. J Crohns Colitis. 2024;18:1283-1291. 10.1093/ECCO-JCC/JJAE031 38506097 PMC11324337

[9] Chirumamilla CS , Fazil MHUT, Perez-Novo C, et al Profiling activity of cellular kinases in migrating T-cells. Methods Mol Biol. 2019;1930:99-113. 10.1007/978-1-4939-9036-8_13/FIGURES/5 30610604

[10] Johnson WE , Li C, Rabinovic A. Adjusting batch effects in microarray expression data using empirical Bayes methods. Biostatistics. 2007;8:118-127. 10.1093/BIOSTATISTICS/KXJ037 16632515

[11] Chen C , Grennan K, Badner J, et al Removing batch effects in analysis of expression microarray data: an evaluation of six batch adjustment methods. PLoS One. 2011;6:e17238. 10.1371/JOURNAL.PONE.0017238 21386892 PMC3046121

[12] Metz KS , Deoudes EM, Berginski ME, et al Coral: clear and customizable visualization of human kinome data. Cell Syst. 2018;7:347-350.e1. 10.1016/J.CELS.2018.07.001 30172842 PMC6366324

[13] Scholman RC , Giovannone B, Hiddingh S, et al Effect of anticoagulants on 162 circulating immune related proteins in healthy subjects. Cytokine. 2018;106:114-124. 10.1016/J.CYTO.2017.10.021 29089178

[14] R Core Team. R: A language and environment for statistical computing computing. R Foundation for Statistical Computing; 2018. https://www.R-project.org/. n.d.

[15] Vossenkämper A , Hundsrucker C, Page K, et al A CD3-specific antibody reduces cytokine production and alters phosphoprotein profiles in intestinal tissues from patients with inflammatory bowel disease. Gastroenterology. 2014;147:172-183. 10.1053/J.GASTRO.2014.03.049 24704524

[16] Danopoulos S , Schlieve CR, Grikscheit TC, Al Alam D. Fibroblast growth factors in the gastrointestinal tract: twists and turns. n.d. 10.1002/dvdy 28198118

[17] Shen L , Li Y, Zhao H. Fibroblast growth factor signaling in macrophage polarization: impact on health and diseases. Front Immunol. 2024;15:1390453. 10.3389/fimmu.2024.1390453 38962005 PMC11219802

[18] Coulthard MG , Morgan M, Woodruff TM, et al Eph/ephrin signaling in injury and inflammation. Am J Pathol. 2012;181:1493-1503. 10.1016/j.ajpath.2012.06.043 23021982

[19] Darling TK , Lamb TJ. Emerging roles for Eph receptors and ephrin ligands in immunity. Front Immunol. 2019;10:1473. 10.3389/fimmu.2019.01473 31333644 PMC6620610

[20] West NR , Hegazy AN, Owens BMJ, et al; Oxford IBD Cohort Investigators. Oncostatin M drives intestinal inflammation and predicts response to tumor necrosis factor-neutralizing therapy in patients with inflammatory bowel disease. Nat Med. 2017;23:579-589. 10.1038/NM.4307 28368383 PMC5420447

[21] Gaujoux R , Starosvetsky E, Maimon n, et al; Israeli IBD research Network (IIRN). Cell-centred meta-analysis reveals baseline predictors of anti-TNFα non-response in biopsy and blood of patients with IBD. Gut. 2019;68:604-614. 10.1136/GUTJNL-2017-315494 29618496 PMC6580771

[22] Digby-Bell JL , Atreya R, Monteleone G, Powell N. Interrogating host immunity to predict treatment response in inflammatory bowel disease. Nat Rev Gastroenterol Hepatol. 2020;17:9-20. 10.1038/S41575-019-0228-5 31767987

[23] Lees CW , Deuring JJ, Chiorean M, et al Prediction of early clinical response in patients receiving tofacitinib in the OCTAVE Induction 1 and 2 studies. Therap Adv Gastroenterol. 2021;14:17562848211054710. 10.1177/17562848211054710 PMC8832332 35154388

[24] Dubinsky MC , Magro F, Steinwurz F, et al Association of C-reactive protein and partial Mayo score with response to tofacitinib induction therapy: results from the ulcerative colitis clinical program. Inflamm Bowel Dis. 2023;29:51-61. 10.1093/IBD/IZAC061 35380664 PMC9825285

[25] Joustra V , Li Yim AYF, van Gennep S, et al Peripheral blood DNA methylation signatures and response to tofacitinib in moderate-to-severe ulcerative colitis. J Crohns Colitis. 2024;18:1179-1189. 10.1093/ECCO-JCC/JJAD129 37526299 PMC11324342

[26] Jang KK , Hudesman D, Jones DR, Loke P, Axelrad JE, Cadwell K; Tofacitinib Working Group. Tofacitinib uptake by patient-derived intestinal organoids predicts individual clinical responsiveness. Gastroenterology. 2024;167:1453-1456.e5. 10.1053/J.GASTRO.2024.07.035 39094749 PMC11581922

[27] Noor NM , Lee JC, Bond S, et al; PROFILE Study Group. A biomarker-stratified comparison of top-down versus accelerated step-up treatment strategies for patients with newly diagnosed Crohn’s disease (PROFILE): a multicentre, open-label randomised controlled trial. Lancet Gastroenterol Hepatol. 2024;9:415-427. 10.1016/S2468-1253(24)00034-7 38402895 PMC11001594

[28] Sancar A , Lindsey-Boltz LA, Ünsal-Kaçmaz K, Linn S. Molecular mechanisms of mammalian DNA repair and the DNA damage checkpoints. Annu Rev Biochem. 2004;73:39-85. 10.1146/ANNUREV.BIOCHEM.73.011303.073723 15189136

[29] Luciani MG , Campregher C, Fortune JM, Kunkel TA, Gasche C. 5-ASA affects cell cycle progression in colorectal cells by reversibly activating a replication checkpoint. Gastroenterology. 2007;132:221-235. 10.1053/J.GASTRO.2006.10.016 17241873 PMC1839818

[30] Ding L , Weygant N, Ding C, Lai Y, Li H. DCLK1 and tuft cells: Immune-related functions and implications for cancer immunotherapy. Crit Rev Oncol Hematol. 2023;191:104118. 10.1016/J.CRITREVONC.2023.104118 37660932

[31] Howitt MR , Lavoie S, Michaud M, et al Tuft cells, taste-chemosensory cells, orchestrate parasite type 2 immunity in the gut. Science. 2016;351:1329-1333. 10.1126/SCIENCE.AAF1648/SUPPL_FILE/AAF1648-HOWITT-SM.PDF 26847546 PMC5528851

[32] Yi J , Bergstrom K, Fu J, et al Dclk1 in tuft cells promotes inflammation-driven epithelial restitution and mitigates chronic colitis. Cell Death Differ. 2019;26:1656-1669. 10.1038/s41418-018-0237-x 30478383 PMC6748088

[33] Melón-Ardanaz E , Veny M, Corraliza AM, et al Understanding the mechanisms underlying the lack of response to Janus kinase inhibition in ulcerative colitis. BioRxiv. 2024. 10.1101/2024.10.17.618410

