# Supplementary material for: Mucosal kinase activity and inflammatory profiles in inflammatory bowel disease, and in relation to tofacitinib response
Source: J Crohns Colitis. 2025 Sep 23;19(10):jjaf174. doi: 10.1093/ecco-jcc/jjaf174 (PMC12597136; doi:10.1093/ecco-jcc/jjaf174)
Supplement: jjaf174_Supplementary_Data [file jjaf174_supplementary_data.zip › Supplement Data/Supplementary figure 1.pdf]

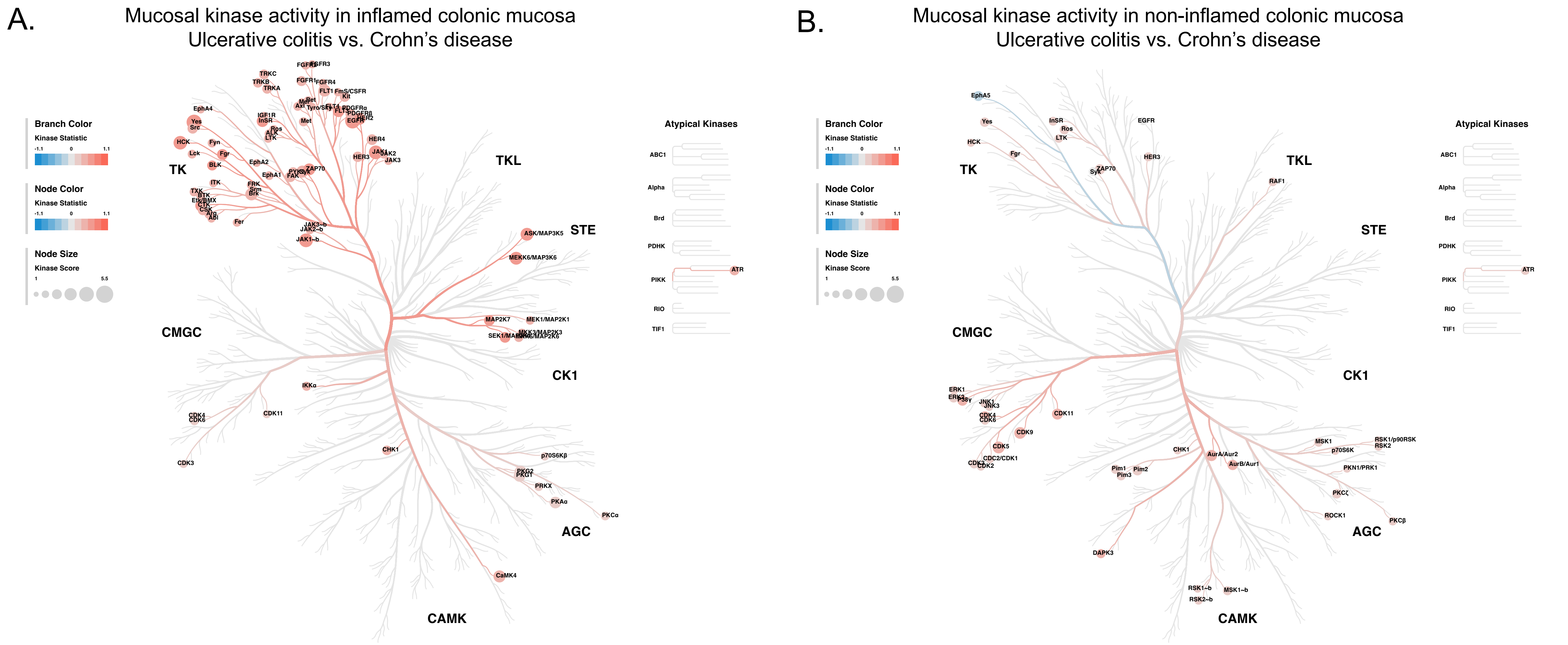
A.
Mucosal kinase activity in inflamed colonic mucosa
Ulcerative colitis vs. Crohn's disease
B.
Mucosal kinase activity in non-inflamed colonic mucosa
Ulcerative colitis vs. Crohn's disease
Branch Color
Kinase Statistic
-1.1
0
1.1
Node Color
Kinase Statistic
-1.1
0
1.1
Node Size
Kinase Score
1
5.5
TK
TKL
STE
CK1
AGC
CAMK
CMGC
Atypical Kinases
ABC1
Alpha
Brd
PDHK
PIKK
RIO
TIF1
ATR
