# Supplementary material for: Mucosal kinase activity and inflammatory profiles in inflammatory bowel disease, and in relation to tofacitinib response
Source: J Crohns Colitis. 2025 Sep 23;19(10):jjaf174. doi: 10.1093/ecco-jcc/jjaf174 (PMC12597136; doi:10.1093/ecco-jcc/jjaf174)
Supplement: jjaf174_Supplementary_Data [file jjaf174_supplementary_data.zip › Supplement Data/Supplementary figure 2.pdf]

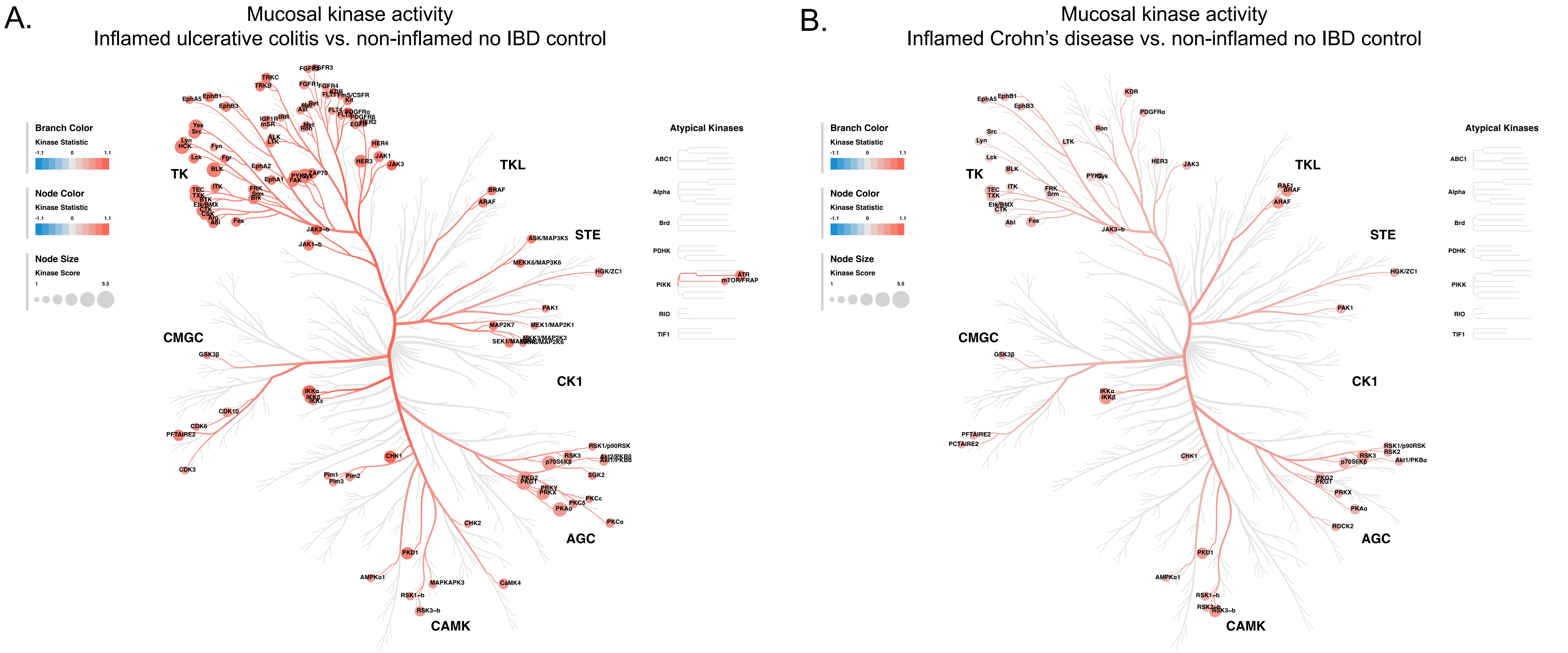
A.
Mucosal kinase activity
Inflamed ulcerative colitis vs. non-inflamed no IBD control
B.
Mucosal kinase activity
Inflamed Crohn's disease vs. non-inflamed no IBD control
Branch Color
Kinase Statistic
-1.1
0
1.1
Node Color
Kinase Statistic
-1.1
0
1.1
Node Size
Kinase Score
1
5.5
TK
TKL
STE
CK1
AGC
CAMK
CMGC
Atypical Kinases
ABC1
Alpha
Brd
PDHK
PIKK
RIO
TIF1
ATR
mTOR/FRAP
BRAF
ARAF
ASK/MAP3K5
MEKK6/MAP3K6
HGK/ZC1
PAK1
MAP2K7
MEK1/MAP2K1
GSK3β
IKKα
IKKβ
CDK10
CDK6
PFTAIRE2
CDK3
CHK1
Pim1
Pim3
Pim2
CHK2
PKD1
AMPKα1
MAPKAPK3
CaMK4
RSK1~b
RSK3~b
RSK1/p90RSK
RSK3
p70S6Kβ
SGK2
PKG2
PKG1
PRKY
PRKX
PKCδ
PKCε
PKAα
PKCα
JAK3~b
JAK1~b
HER4
HER3
JAK1
JAK3
EphA5
EphB1
EphB3
Yes
Src
Lyn
HCK
Fyn
Lck
Fgr
BLK
EphA2
EphA1
ITK
TEC
TXK
FRK
Srm
Brk
Fes
Abl
TRKC
TRKB
FGFR1
FGFR4
KDR
IGF1R
InSR
ALK
LTK
FLT3
PDGFRα
PDGFRβ
EGFR
HER2
ZAP70
FAK
KDR
PDGFRα
Ron
LTK
PYK2
Syk
RAF1
BRAF
ARAF
RSK2
Akt1/PKBα
ROCK2
PCTAIRE2
RSK2~b
