# Supplementary material for: Mucosal kinase activity and inflammatory profiles in inflammatory bowel disease, and in relation to tofacitinib response
Source: J Crohns Colitis. 2025 Sep 23;19(10):jjaf174. doi: 10.1093/ecco-jcc/jjaf174 (PMC12597136; doi:10.1093/ecco-jcc/jjaf174)
Supplement: jjaf174_Supplementary_Data [file jjaf174_supplementary_data.zip › Supplement Data/Supplementary figure 3.pdf]

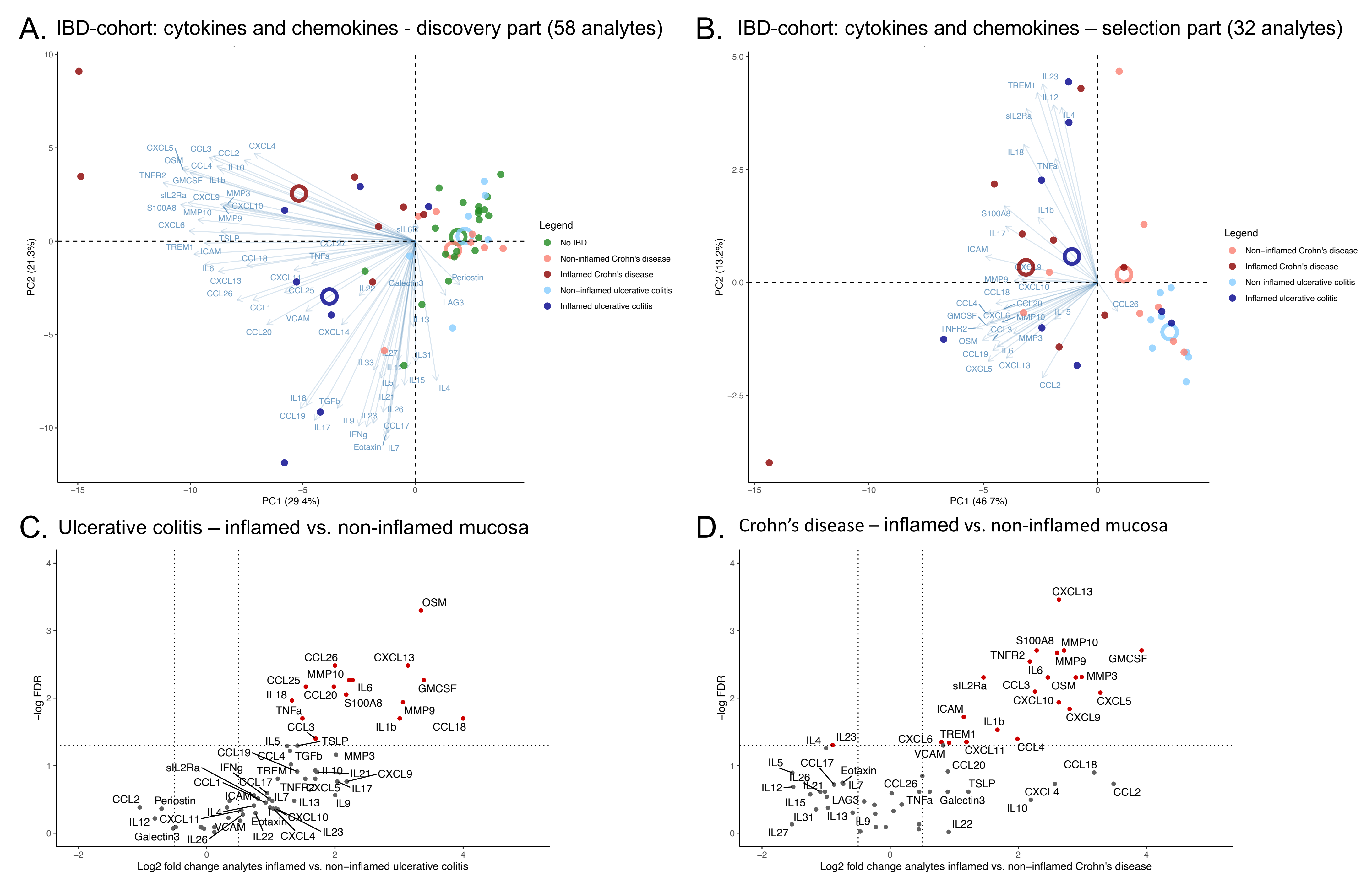

A. IBD-cohort: cytokines and chemokines - discovery part (58 analytes)
B. IBD-cohort: cytokines and chemokines – selection part (32 analytes)
C. Ulcerative colitis – inflamed vs. non-inflamed mucosa
D. Crohn's disease – inflamed vs. non-inflamed mucosa
PC1 (29.4%)
PC2 (21.3%)
PC1 (46.7%)
PC2 (13.2%)
Legend
No IBD
Non-inflamed Crohn's disease
Inflamed Crohn's disease
Non-inflamed ulcerative colitis
Inflamed ulcerative colitis
CXCL5 CCL3 CCL2 CXCL4 OSM CCL4 IL10 TNFR2 GMCSF IL1b sIL2Ra CXCL9 MMP3 CXCL10 S100A8 MMP10 MMP9 CXCL6 TSLP sIL6R TREM1 ICAM CCL27 CCL18 TNFa IL6 CXCL13 CXCL11 CCL26 CCL25 IL22 Galectin3 Periostin CCL1 VCAM LAG3 CCL20 CXCL14 IL13 IL27 IL31 IL33 IL12 IL5 IL15 IL4 IL21 IL18 TGFb IL26 CCL19 IL23 IL17 IL9 CCL17 IFNg Eotaxin IL7
IL23 TREM1 IL12 IL4 sIL2Ra IL18 TNFa S100A8 IL1b IL17 ICAM CXCL9 MMP9 CXCL10 CCL18 CCL26 CCL4 CCL20 GMCSF CXCL6 MMP10 IL15 TNFR2 CCL3 OSM MMP3 IL6 CCL19 CXCL13 CXCL5 CCL2
-log FDR
Log2 fold change analytes inflamed vs. non−inflamed ulcerative colitis
Log2 fold change analytes inflamed vs. non−inflamed Crohn's disease
OSM CCL26 CXCL13 MMP10 CCL25 IL6 GMCSF IL18 CCL20 S100A8 TNFa MMP9 IL1b CCL18 CCL3 IL5 TSLP CCL19 CCL4 TGFb MMP3 sIL2Ra IFNg TREM1 IL10 IL21 CXCL9 CCL1 CCL17 TNFR2 CXCL5 IL17 ICAM IL7 IL13 IL9 CCL2 Periostin CXCL10 IL4 IL12 CXCL11 VCAM Eotaxin Galectin3 IL26 IL22 CXCL4 IL23
CXCL13 S100A8 MMP10 TNFR2 MMP9 GMCSF IL6 MMP3 sIL2Ra CCL3 OSM CXCL5 CXCL10 CXCL9 ICAM IL1b IL4 IL23 CXCL6 TREM1 CCL4 CXCL11 VCAM CCL20 CCL18 IL5 CCL17 Eotaxin IL26 IL7 IL12 IL21 CCL26 TSLP CCL2 LAG3 CXCL4 IL15 TNFa Galectin3 IL10 IL31 IL13 IL9 IL22 IL27
