# Supplementary material for: Mucosal kinase activity and inflammatory profiles in inflammatory bowel disease, and in relation to tofacitinib response
Source: J Crohns Colitis. 2025 Sep 23;19(10):jjaf174. doi: 10.1093/ecco-jcc/jjaf174 (PMC12597136; doi:10.1093/ecco-jcc/jjaf174)
Supplement: jjaf174_Supplementary_Data [file jjaf174_supplementary_data.zip › Supplement Data/Supplementary tables 1-2, 8-9, 11 and 14 and supplementary figure legends.docx]

**SUPPLEMENTARY MATERIAL**

**Mucosal Kinase Activity and Inflammatory Profiles in Inflammatory Bowel Disease, and in Relation with Tofacitinib Response**

**Authors**

Eelco C. Brand^1,2^,

Britt Roosenboom^3^,

Lisanne Lutter^1,2^,

Bea Malvar Fernandez^4^,

Savithri Rangarajan^5^,

Elly van Koolwijk^6^,

Sara van Gennep^7^,

Geert R.A.M. D’Haens^7^,

Ellen G. van Lochem^6^,

Carmen S. Horjus Talabur Horje^3^,

Kris A. Reedquist^8,#^,

Femke van Wijk^2,#^,

Bas Oldenburg^1,#^

*^#^Contributed equally and share senior-authorship*

**Affiliations**

1. Department of Gastroenterology and Hepatology, University Medical Center Utrecht, Utrecht University, Utrecht, The Netherlands.
2. Center for Translational Immunology, University Medical Center Utrecht, Utrecht University, Utrecht, The Netherlands.
3. Crohn & Colitis Center Rijnstate, Department of Gastroenterology and Hepatology, Rijnstate Hospital, Arnhem, the Netherlands.
4. Galicia Sur Health Research Institute, Rheumatology and Immune-mediated Diseases Group, Vigo, Spain. Previously affiliated with the Center for Translational Immunology, University Medical Center Utrecht, Utrecht University, Utrecht, The Netherlands.
5. PamGene International B.V., 's-Hertogenbosch, the Netherlands.
6. Department of Microbiology and Immunology, Rijnstate Hospital, Arnhem, the Netherlands.
7. Department of Gastroenterology and Hepatology, Amsterdam UMC, Amsterdam, the Netherlands.
8. Independent researcher. Previously affiliated with the Center for Translational Immunology, University Medical Center Utrecht, Utrecht University, Utrecht, The Netherlands.

**SUPPLEMENTARY FIGURE LEGENDS**

**Supplementary figure 1. Colonic mucosal kinase activity comparing ulcerative colitis to Crohn’s disease.** Predicted kinase activity is plotted on the human kinome tree^1^. A kinase statistic > 0 depicts kinases that are more active in the colonic mucosa of UC patients (N=16) compared to Crohn’s disease patients (N=16). Only predicated kinases with a median kinase score > 1.3 are plotted. A) Kinase activity comparing inflamed mucosa, and B) comparing non-inflamed mucosa.

**Supplementary figure 2. Colonic mucosal kinase activity comparing inflamed mucosa of ulcerative colitis to non-inflamed mucosa of non-IBD controls, and inflamed mucosa of Crohn’s disease tot non-inflamed mucosa of non-IBD controls.** Predicted kinase activity is plotted on the human kinome tree^1^. A kinase statistic > 0 depicts kinases that are more active in the inflamed colonic mucosa of UC patients (N=16) (panel A) or in the inflamed colonic mucosa of Crohn’s disease patients (N=16) (panel B) compared to the non-inflamed colonic mucosa of non-IBD controls (N=4). Only predicated kinases with a median kinase score > 1.3 are plotted.

**Supplementary figure 3. Colonic mucosal cytokine and chemokine levels in non-stimulated 24-hour explant culture.** A) Principal component analyses of cytokine and chemokine levels corrected for biopsy mass of 58 analytes measured with Luminex in the supernatant of non-stimulated 24-hour colonic mucosa explant cultures. For the cytokine/chemokine measurement discovery part of the IBD cohort, 8 UC patients (7 inflamed and 6 non-inflamed samples [one inflamed and one non-inflamed sample from different patients did not pass quality control, and for one non-inflamed sample the biopsy mass was missing]), and 7 CD patients (7 inflamed and 7 non-inflamed samples) were included. In addition, 5 colonic biopsies per participant from 4 non-IBD individuals served as controls (20 samples) . Each small dot represents one sample, each open circle depicts the centroid per group. All analytes analyzed are depicted with an arrow showing their loading for the PCA plot. PC1 and PC2 (explained variance between brackets) are plotted on the x- and y-axis respectively. Inflamed samples are clearly separated from non-inflamed samples (PERMANOVA FDR < 0.05 for inflamed vs. non-inflamed comparisons, supplementary table 8). B) PCA-plot depicting the cytokine and chemokine profiles corrected for biopsy mass based on a 32-analyte panel for 8 CD patients (8 inflamed and 8 non-inflamed samples) and 8 UC patients (8 inflamed and 8 non-inflamed samples) from the cytokine/chemokine measurement selection part of the IBD cohort. Inflamed samples are clearly separated from non-inflamed samples (PERMANOVA FDR <0.05 comparing inflamed CD vs non-inflamed CD and inflamed UC vs non-inflamed UC, supplementary table 9). C-D) Volcano plots showing the log_2_ fold change and -log_10_ false discovery rate for 55 analytes measured in the cytokine/chemokine measurement discovery part of the IBD cohort comparing inflamed to non-inflamed mucosal biopsies for C) UC and D) CD. Red dots represent analytes that have a log_2_ fold change >0.5 and an FDR <0.05. All analytes with a log_2_ fold change >0.5 or <0.5 are denoted.

CD, Crohn’s disease; FDR, false discovery rate; IBD, inflammatory bowel disease; PC, principal component; PCA, principal component analyses; PERMANOVA, permutational multivariate analysis of variance; UC, ulcerative colitis.

**Supplementary figure 4. Cytokine and chemokine levels corrected for biopsy mass in a 24 hour explant culture of colonic mucosal biopsies of ulcerative colitis patients before start of tofacitinib treatment.** Radar plot showing the box-cox transformed mean values of 32 analytes measured in the supernatant of a 24-hour inflamed colonic mucosal explant culture of 6 non-responders, and 4 responders (for 1 responder the biopsy was cultured >24 hours, and one sample did not pass quality control) to tofacitinib. Colonic samples were collected before the start of treatment. The false discovery rate comparing the levels of the cytokines and chemokines between responders and non-responders was > 0.78 for all comparisons, i.e. no statistically significant differences in the spontaneous produced cytokines and chemokines in association with tofacitinib were found. **Supplementary figure 5. Colonic mucosal kinase activity after 8 weeks of tofacitinib treatment comparing responders to non-responders.** Predicted kinase activity is plotted on the human kinome tree^1^. A kinase statistic < 0 depicts kinases that are less active in the colonic mucosa of UC patients that responded to tofacitinib treatment (N=8) compared to those who did not respond (N=8) after 8 weeks of treatment. Only predicated kinases with a median kinase score > 1.3 are plotted.

**SUPPLEMENTARY TABLES**

| Supplementary table 1. Quality control measures for kinase activity measurement | | | |
| --- | --- | --- | --- |
|  | **IBD cohort** | **Tofacitinib cohort 1** | **Tofacitinib cohort 2** |
| Protein Tyrosine Kinase (PTK) measurements | | | |
| 99th percentile peptide signal strength^a^, (arbitrary units) | 3231 | 3315 | 1986 |
| Peptides that passed quality control^b^, N (%) | 154 (79.0%) | 159 (81.5%) | 150 (76.9%) |
| Serine / Threonine Kinase (STK) measurements | | | |
| 99th percentile peptide signal strength^a^, (arbitrary units) | 3565 | 1839 | 3149 |
| Peptides that passed quality control^c^, N (%) | 84 (59.2%) | 99 (69.7%) | 95 (66.9%) |
| N, number; p99, 99th percentile.  ^a^Signal strength between 2000 and 4000 is considered good, between 1000 and 2000 is considered fair, and below 1000 is considered poor.  ^b^A total number of 195 peptides is taken into account. Only peptides that show an increase in signal over time in ≥ 25% of the arrays are included.  ^c^A total number of 142 peptides is taken into account. Only peptides that have a positive signal at the last cycle are included. | | | |

| Supplementary table 2. Analytes selected for multiplex protein analyses. | | | | | | |
| --- | --- | --- | --- | --- | --- | --- |
| Analyte | Synonym | Full name | Discovery panel IBD cohort | Selection panel IBD cohort & tofacinib cohort 1 | JAK-signalling involved^2–5^ | Family or class^6–13^ |
| IL1b |  | Interleukin 1 beta | Yes | Yes |  | Interleukin-1 family |
| IL4 |  | Interleukin 4 | Yes | Yes | JAK1 & JAK3 | Interleukin-2 family |
| IL6 |  | Interleukin 6 | Yes | Yes | JAK1 & JAK2 & TYK2 | Interleukin-6 family |
| IL10 |  | Interleukin 10 | Yes | Yes | JAK1 & JAK3 & TYK2 | Interleukin-10 family |
| IL12 | IL12 p70 | Interleukin 12 | Yes | Yes | JAK2 & TYK2 | Interleukin-12 family |
| IL15 |  | Interleukin 15 | Yes | Yes | JAK1 & JAK3 | Interleukin-2 family |
| IL17 | IL17A | Interleukin 17 | Yes | Yes |  | Interleukin-17 family |
| IL18 |  | Interleukin 18 | Yes | Yes |  | Interleukin-1 family |
| IL23 | IL23 p19 | Interleukin 23 | Yes | Yes | JAK2 & TYK2 | Interleukin-12 family |
| TNFa |  | Tumor Necrosis Factor alpha | Yes | Yes |  | TNF superfamily |
| OSM |  | Oncostatin M | Yes | Yes | JAK1 & JAK2 & TYK2 | Interleukin-6 family |
| CCL2 | MCP1 | C-C motif Chemokine Ligand 2 / Monocyte Chemotactic Protein 1 | Yes | Yes |  | Chemokine family - C-C Subfamily |
| CCL3 | MIP1a | C-C motif Chemokine Ligand 3 / Macrophage Inflammatory Protein 1 alpha | Yes | Yes |  | Chemokine family - C-C Subfamily |
| CCL4 | MIP1b | C-C motif Chemokine Ligand 4 / Macrophage Inflammatory Protein 1 beta | Yes | Yes |  | Chemokine family - C-C Subfamily |
| CCL18 | PARC | C-C motif Chemokine Ligand 18 / Pulmonary and Activation Regulated Chemokine | Yes | Yes |  | Chemokine family - C-C Subfamily |
| CCL19 | MIP3b | C-C motif Chemokine Ligand 19 / Macrophage Inflammatory Protein 3 beta | Yes | Yes |  | Chemokine family - C-C Subfamily |
| CCL20 | MIP3a | C-C motif Chemokine Ligand 20 / Macrophage Inflammatory Protein 3 alpha | Yes | Yes |  | Chemokine family - C-C Subfamily |
| CCL26 | Eotaxin3 | C-C motif Chemokine Ligand 26 / Eotaxin 3 | Yes | Yes |  | Chemokine family - C-C Subfamily |
| CXCL5 | ENA78 | Chemokine (C-X-C motif) ligand 5 / Epithelial-derived Neutrophil-Activating Protein 78 | Yes | Yes |  | Chemokine family - CXC Subfamily |
| CXCL6 | GCP2 | Chemokine (C-X-C motif) ligand 6 / Granulocyt Chemotactic Protein 2 | Yes | Yes |  | Chemokine family - CXC Subfamily |
| CXCL9 | MIG | Chemokine (C-X-C motif) ligand 9 / Monokine induced by Interferon Gamma | Yes | Yes |  | Chemokine family - CXC Subfamily |
| CXCL10 | IP10 | Chemokine (C-X-C motif) ligand 10 / Interferon gamma induced Protein 10 | Yes | Yes |  | Chemokine family - CXC Subfamily |
| CXCL13 | BLC | Chemokine (C-X-C motif) ligand 13 / B Lymphocyte Chemoattractant | Yes | Yes |  | Chemokine family - CXC Subfamily |
| GMCSF |  | Granulocyte-Macrophage Colony-Stimulating Factor | Yes | Yes | JAK2 & JAK2 | Hematopoietic family |
| ICAM | sCD54 | Intercellular Adhesion Molecule 1 / soluble CD54 | Yes | Yes |  | Immunoglobulin superfamily - Integrin binding receptors |
| MMP3 | Stromelysin 1 | Matrix Metalloproteinase 3 / Stromelysin 1 | Yes | Yes |  | Matrix metalloproteinases |
| MMP9 |  | Matrix Metalloproteinase 9 | Yes | Yes |  | Matrix metalloproteinases |
| MMP10 |  | Matrix metalloproteinase 10 | Yes | Yes |  | Matrix metalloproteinases |
| S100A8 |  | S100 calcium-binding protein 8 [calprotectin when in combination with S100A9] | Yes | Yes |  | S100 calcium-binding proteins |
| TREM1 | CD354 | Triggering receptor expressed on myeloid cells 1 TREM1 / CD354 | Yes | Yes |  | Immunoglobulin superfamily |
| TNFR2 |  | Tumor Necrosis Factor Receptor 2 | Yes | Yes |  | TNF receptor superfamily |
| sIL2Ra | sCD25 | soluble interleukin 2 receptor alpha / soluble CD25 | Yes | Yes |  | IL2 receptor family |
| IL2^a^ |  | Interleukin 2 | Yes |  | JAK1 & JAK3 | Interleukin-2 family |
| IL5 |  | Interleukin 5 | Yes |  | JAK2 & JAK2 | Hematopoietic family |
| IL7 |  | Interleukin 7 | Yes |  | JAK1 & JAK3 | Interleukin-2 family |
| IL9 |  | Interleukin 9 | Yes |  | JAK1 & JAK3 | Interleukin-2 family |
| IL13 |  | Interleukin 13 | Yes |  | JAK1 & JAK2 & TYK2 | Interleukin-2 family |
| IL21 |  | Interleukin 21 | Yes |  | JAK1 & JAK3 | Interleukin-2 family |
| IL22 |  | Interleukin 22 | Yes |  | JAK1 & JAK3 & TYK2 | Interleukin-10 family |
| IL26 |  | Interleukin 26 | Yes |  | JAK1 & JAK3 & TYK2 | Interleukin-10 family |
| IL27 |  | Interleukin 27 | Yes |  | JAK1 & JAK2 & TYK2 | Interleukin-12 family |
| IL31 |  | Interleukin 31 | Yes |  | JAK1 & JAK2 & TYK2 | Interleukin-6 family |
| IL33 |  | Interleukin 33 | Yes |  |  | Interleukin-1 family |
| IFNg |  | Interferon gamma | Yes |  | JAK1 & JAK2 (& TYK2) | Interferon family |
| TSLP |  | Thymic Stromal Lymphopoietin | Yes |  | JAK1 & JAK2 | Interleukin-2 family |
| LAG3 |  | Lymphocyte activation gene 3 protein | Yes |  |  | Immunoglobulin superfamily - Inhibitory receptors |
| TGFb | LAP | Transforming Growth Factor beta / Latency-Associated Peptide | Yes |  |  | TGF superfamily |
| CCL1 | I309 | C-C motif Chemokine Ligand 1 / I-309 | Yes |  |  | Chemokine family - C-C Subfamily |
| Eotaxin | CCL11 | Eotaxin / C-C motif Chemokine Ligand 11 | Yes |  |  | Chemokine family - C-C Subfamily |
| CCL17 | TARC | C-C motif Chemokine Ligand 17 / Thymus and Activation Regulated Chemokine | Yes |  |  | Chemokine family - C-C Subfamily |
| CCL25 | TECK | C-C motif Chemokine Ligand 25 / Thymus-Expressed Chemokine | Yes |  |  | Chemokine family - C-C Subfamily |
| CCL27 | CTACK | C-C motif Chemokine Ligand 27 / Cutaneous T cell Attracting Chemokine | Yes |  |  | Chemokine family - C-C Subfamily |
| CCL28^b^ | MEC | C-C motif Chemokine Ligand 28 / Mucosa-associated Epithelial Chemokine | Yes |  |  | Chemokine family - C-C Subfamily |
| CXCL4 | PF4 | Chemokine (C-X-C motif) ligand 4 / Platelet Factor 4 | Yes |  |  | Chemokine family - CXC Subfamily |
| IL8^b^ | CXCL8 | Interleukin 8 / Chemokine (C-X-C motif) ligand 8 | Yes |  |  | Chemokine family - CXC Subfamily |
| CXCL11 | I-TAC | Chemokine (C-X-C motif) ligand 11 / Interferon inducible T-cell alpha chemoattractant | Yes |  |  | Chemokine family - CXC Subfamily |
| CXCL14 | BRAK / MIP2g | Chemokine (C-X-C motif) ligand 14 / Breast and Kidney expressed chemokine / Macrophage Inflammatory Protein 2 gamma | Yes |  |  | Chemokine family - CXC Subfamily |
| Periostin | OSF2 | Periostin / Osteoblast-specific factor 2 | Yes |  |  | Extracellular matrix proteins |
| VCAM | sCD106 | Vascular Cell Adhesion Molecule 1 / soluble CD106 | Yes |  |  | Immunoglobulin superfamily - Integrin binding receptors |
| MMP1^b^ |  | Matrix Metalloproteinase 1 | Yes |  |  | Matrix metalloproteinases |
| TIMP1^b^ |  | Tissue Inhibitor of Metalloproteinases 1 | Yes |  |  | Inhibitors of matrix metalloproteinases |
| sIL6R | sCD126 | soluble interleukin 6 receptor / soluble CD126 | Yes |  |  | IL6 receptor family |
| Galectin1^b^ |  | Galectin 1 | Yes |  |  | Galectins |
| Galectin3 |  | Galectin 3 | Yes |  |  | Galectins |
| Galectin9^b^ |  | Galectin 9 | Yes |  |  | Galectins |
| JAK, janus kinase; TYK2, tyrosine kinase 2.  ^a^IL2 was excluded from the analyses because in ≥30% of samples <20 beads were measured.  ^b^CCL28, IL8, MMP1, TIMP1, galectin1 and galectin9 were excluded from the analyses because >30% of measurements were outside of the limits of quantification, mostly above the upper limit of detection except for CCL28. | | | | | | |

**Supplementary tables 3 – 7**

These tables are displayed in the supplementary excel file.

| Supplementary table 8. PERMANOVA results – cytokine/chemokine analyses. IBD cohort – discovery phase (58 analytes) | |
| --- | --- |
| Comparison | **False discovery rate (FDR)** |
| *Inflamed ulcerative colitis vs non-inflamed ulcerative colitis* | *0.00200* |
| *Inflamed Crohn's disease vs non-inflamed Crohn's disease* | *0.00617* |
| *Inflamed Crohn's disease vs non-inflamed ulcerative colitis* | *0.00150* |
| *Non-inflamed Crohn's disease vs inflamed ulcerative colitis* | *0.00150* |
| *No IBD vs inflamed Crohn's disease* | *0.00050* |
| *No IBD vs inflamed ulcerative colitis* | *0.00050* |
| Inflamed Crohn's disease vs inflamed ulcerative colitis | 0.07799 |
| No IBD vs non-inflamed ulcerative colitis | 0.07799 |
| No IBD vs non-inflamed Crohn's disease | 0.36363 |
| Non-inflamed Crohn's disease vs non-inflamed ulcerative colitis | 0.44976 |
| PERMANOVA results based on the Euclidean distance matrix of log-normalized cytokine/chemokine levels corrected for biosy mass in the IBD cohort – discovery phase (58 analytes).  This analyses include 7 non-inflamed CD and 7 inflamed CD samples for 7 CD participants, 6 non-inflamed UC and 7 inflamed UC samples from 7 UC participants, and 20 non-inflamed no IBD samples from 4 no IBD participants. Cytokine and chemokine levels are corrected for biopsy mass and based on 24-hour explant culture of colonic mucosal biopsies.  Statistically significant (FDR < 0.05) results are depicted in an *italic font*.  CD, Crohn’s disease; FDR, false discovery rate; IBD, inflammatory bowel disease; PERMANOVA, permutational multivariate analysis of variance; UC, ulcerative colitis. | |

| Supplementary table 9. PERMANOVA results – cytokine/chemokine analyses. IBD cohort – selection phase (32 analytes) | |
| --- | --- |
| Comparison | **False discovery rate (FDR)** |
| *Inflamed ulcerative colitis vs non-inflamed ulcerative colitis* | *0.02700* |
| *Inflamed Crohn's disease vs non-inflamed Crohn's disease* | *0.03280* |
| *Non-inflamed Ulcerative colitis vs Inflamed Crohn's disease* | *0.00180* |
| Inflamed ulcerative colitis vs non-inflamed Crohn's disease | 0.21058 |
| Inflamed ulcerative colitis vs Inflamed Crohn's disease | 0.29787 |
| Non-inflamed ulcerative colitis vs non-inflamed Crohn's disease | 0.21058 |
| PERMANOVA results based on the Euclidean distance matrix of log-normalized cytokine/chemokine levels corrected for biosy mass in the IBD cohort – selection phase (32 analytes).  This analyses include 8 non-inflamed CD and 8 inflamed CD samples from 8 CD participants, and 8 non-inflamed UC and 8 inflamed UC samples from 8 UC participants. Cytokine and chemokine levels are corrected for biopsy mass and based on 24-hour explant culture of colonic mucosal biopsies.  Statistically significant (FDR < 0.05) results are depicted in an *italic font*.  CD, Crohn’s disease; FDR, false discovery rate; IBD, inflammatory bowel disease; PERMANOVA, permutational multivariate analysis of variance; UC, ulcerative colitis. | |

**Supplementary tables 10**

These tables are displayed in the supplementary excel file.

| Supplementary table 11. Clinical, biochemical and endoscopic data according to response to tofacitinib in the tofacitinib cohorts. | | | | |
| --- | --- | --- | --- | --- |
|  | **Tofacitinib cohort 1** | | **Tofacitinib cohort 2** | |
|  | **Non-responder**  **(N=6)** | **Responder**  **(N=6)** | **Non-responder**  **(N=8)** | **Responder**  **(N=8)** |
| *Baseline* |  |  |  |  |
| Baseline SCCAI^a^ | - | - | 11.5 (10, 13) | 8 (6.5, 12.3) |
| Baseline CRP level  Days between measurement and biopsy  Missing | 14 (10, 22)  0 (0, 5)  1 | 3 (2.1, 11)  6 (-7.5, 17.3)  - | 30.5 (0.5, 52)  4 (0, 6.3)  - | 2.3 (0.3, 25.6)  6.5 (6.5, 12.5)  - |
| Baseline fecal calprotectin level  Days between measurement and biopsy  Missing | 1710 (1540, 1830)  20.5 (7.3, 30.8)  - | 1300 (480, 2130)  16 (2.5, 25.8)  - | 2300 (835, 3880)  5 (3.5, 8.8)  2 | 4820 (2620, 6000)  12 (6.5, 13.5)  1 |
| Baseline Mayo Endoscopic Score | 2 (2, 2.8) | 2 (2, 2) | 3 (3, 3) | 2.5 (2, 3) |
| *Follow-up* |  |  |  |  |
| Follow-up moment | 3-months | 3-months | 8-weeks | 8-weeks |
| Follow-up SCCAI^a^ | - | - | 5.5 (2.5, 7.5) | 2 (1, 2) |
| Follow-up CRP level^b^  Days between measurement and follow-up^c^  Missing | 23.2 (6, 62)  -1.8 (-4.9, 7.3)  2 | 0.7 (0.6, 5.6)  -4.5 (-7.5, 11.5)  1 | 6.15 (3.2, 16)  0  - | 0.8 (0.4, 1.8)  0  - |
| Follow-up fecal calprotectin level^b^  Days between measurement and follow-up^c^  Missing | 618 (324, 1550)  15.8 (5.8, 24.8)  2 | 76 (62.8, 101)  0.5 (-14.8, 16.3)  2 | 1830 (506, 3030)  0  - | 56.5 (16, 227)  0  - |
| Follow-up Mayo Endoscopic Score^b^  Days between measurement and follow-up^c^  Missing | 2, 3^d^  -27.5, 0^d^  4 | 0, 3^d^  -13.5, -12.5^d^  4 | 3 (2.8, 3)  -  - | 1 (1, 1)  -  - |
| Tofacitinib stopped before follow-up | 4 (66.7%) | 0 | 0 | 0 |
| Colectomy before follow-up | 2 (33.3%) | 0 | 0 | 0 |
| CRP, C reactive protein; N, number of participants; SCCAI, short clinical colitis activity index.  ^a^The SCCAI was not collected in tofacitinib cohort 1.  ^b^Only CRP-levels, fecal calprotectin-levels and Mayo endoscopic subscores that were within one month of the 3-months follow-up or the stopmoment of tofacitinib are reported for tofacitinib cohort 1.  ^c^For tofacitinib cohort 1, this is either 3-months or the stop moment of tofacitinib.  ^d^Only minimum and maximum displayed since this is only available for 2 patients. The patient with a Mayo endoscopic score of 3 in the responder group in Tofacitinib cohort 1 went from a pancolitis Mayo score 3 to only 4cm of inflammation, which was Mayo score 3. | | | | |

**Supplementary tables 12 - 13**

These tables are displayed in the supplementary excel file.

| Supplementary table 14. Mucosal kinase activity associated with response to tofacitinib | | | | | |
| --- | --- | --- | --- | --- | --- |
|  |  | Responders vs. non-responders to tofacitinib  Tofacitinib cohort 1 (N=12) | | Responders vs. non-responders to tofacitinib  Tofacitinib cohort 2 (N=16) | |
| Kinase Name | Kinase Family | Median Final score | Median Kinase Statistic | Median Final score | Median Kinase Statistic |
| *Predicted protein tyrosine kinase (PTK) activity* | | | | | |
| JAK1~b | JakA | 3.10380416 | 0.4132689 |  |  |
| BTK | Tec | 3.08830984 | 0.31350269 |  |  |
| ITK | Tec | 2.70952019 | 0.24161419 |  |  |
| MEKK6/MAP3K6 | STE11 | 2.26472055 | 0.42090564 |  |  |
| ASK/MAP3K5 | STE11 | 2.26472055 | 0.42090564 |  |  |
| FLT1 | VEGFR | 2.25181197 | 0.29290752 |  |  |
| EphA5 | Eph | 1.95366213 | 0.38535234 |  |  |
| SEK1/MAP2K4 | STE7 | 1.81633164 | 0.31818625 |  |  |
| MAP2K7 | STE7 | 1.66154351 | 0.31540746 |  |  |
| EGFR | EGFR | 1.60296426 | 0.2202881 |  |  |
| PDGFR[beta] | PDGFR | 1.57603363 | 0.25857275 |  |  |
| MEK1/MAP2K1 | STE7 | 1.52958951 | 0.31381164 |  |  |
| Syk | Syk |  |  | 2.277366 | 0.194003 |
| BLK | Src |  |  | 1.790807 | 0.217355 |
| Src | Src |  |  | 1.767919 | 0.205357 |
| Etk/BMX | Tec |  |  | 1.718149 | 0.213854 |
| FAK1 | Fak |  |  | 1.667481 | 0.199314 |
| CTK | Csk |  |  | 1.549751 | 0.201125 |
| Ros | Sev |  |  | 1.52308 | 0.290402 |
| Lyn | Src |  |  | 1.335208 | 0.220635 |
| *Predicted serine/threonine kinase (STK) activity* | | | | | |
| Nek2 | NEK | 3.66554625 | 0.706197684 |  |  |
| p38[gamma] | MAPK | 3.40230481 | 0.547127048 |  |  |
| ICK | RCK | 2.70952019 | 0.614302853 |  |  |
| ERK5 | MAPK | 2.40671393 | 0.368548639 |  |  |
| AlphaK1 | Alpha | 2.3378703 | 0.546109507 |  |  |
| ERK2 | MAPK | 2.10039834 | 0.343709378 |  |  |
| DCAMKL1 | DCAMKL | 2.07263648 | 0.470174835 | 1.448463 | 0.503961 |
| CDKL2 | CDKL | 1.99370961 | 0.534239575 |  |  |
| ERK1 | MAPK | 1.89907832 | 0.292776824 |  |  |
| ATR | PIKK | 1.71778587 | 0.341231853 | 1.404945 | 0.406637 |
| AurA/Aur2 | AUR | 1.6466609 | 0.393469341 |  |  |
| JNK1 | MAPK | 1.55783391 | 0.268413142 |  |  |
| JNK3 | MAPK | 1.55783391 | 0.268413142 |  |  |
| CDK2 | CDK | 1.48881207 | 0.291233099 |  |  |
| MAPK14 | MAPK | 1.48243465 | 0.285527594 |  |  |
| CHK1 | CAMKL | 1.47149466 | 0.320922966 |  |  |
| CDK3 | CDK | 1.3086002 | 0.262658144 |  |  |
| p70S6K[beta] | RSK |  |  | 4.251812 | 0.694472 |
| PKG2 | PKG |  |  | 4.221849 | 0.674693 |
| PKG1 | PKG |  |  | 4.19382 | 0.644687 |
| PKA[alpha] | PKA |  |  | 4.055517 | 0.585738 |
| PRKX | PKA |  |  | 3.966576 | 0.634674 |
| IKK[alpha] | IKK |  |  | 3.356547 | 0.930983 |
| CaMK4 | CAMK1 |  |  | 2.820448 | 0.687167 |
| DYRK1A | DYRK |  |  | 2.534788 | -0.71194 |
| PFTAIRE2 | CDK |  |  | 2.376751 | 0.700246 |
| PKC[alpha] | PKC |  |  | 2.356547 | 0.517632 |
| MAPKAPK3 | MAPKAPK |  |  | 2.283329 | 0.477658 |
| SGK2 | SGK |  |  | 2.067729 | 0.50168 |
| ROCK1 | DMPK |  |  | 2.017729 | -0.47584 |
| CDK10 | CDK |  |  | 1.994305 | 0.534545 |
| PRKY | PKA |  |  | 1.920385 | 0.513459 |
| PKC[delta] | PKC |  |  | 1.867484 | 0.475563 |
| MAPKAPK2 | MAPKAPK |  |  | 1.816673 | 0.45966 |
| CK1[alpha] | CK1 |  |  | 1.801123 | 0.506082 |
| CHK2 | RAD53 |  |  | 1.757657 | 0.468526 |
| PKC[gamma] | PKC |  |  | 1.715885 | 0.403725 |
| mTOR/FRAP | PIKK |  |  | 1.702458 | 0.523611 |
| PKD1 | PKD |  |  | 1.645738 | 0.524385 |
| Pim1 | PIM |  |  | 1.486889 | 0.399816 |
| AMPK[alpha]1 | CAMKL |  |  | 1.422508 | 0.46721 |
| HGK/ZC1 | STE20 |  |  | 1.399344 | 0.515662 |
| PKC[epsilon] | PKC |  |  | 1.381116 | 0.407909 |
| IKK[beta] | IKK |  |  | 1.348722 | 0.430237 |
| N, number of ulcerative colitis patients.  Predicted colonic mucosal kinase activity before treatment in association with response to tofactinib in UC patients. In tofacitinib cohort 1, 6 responders are compared to 6 non-responders for the protein tyrosine kinase (PTK) analyses and due to a technical error 5 responders are compared to 6 non-responders for the serine/threonine kinase (STK) analyses. In tofacitinib cohort 2, 8 responders are compared to 8 non-responders.  Only kinases with a median final score >1.3 are displayed, a complete overview can be found in supplementary table 10 and 13. A kinase statistic >0 depicts kinases that are more active in the colonic mucosa of responders compared to non-responders. | | | | | |

**Supplementary tables 15 - 21**

These tables are displayed in the supplementary excel file.

**SUPPLEMENTARY REFERENCES**

1. Metz KS., Deoudes EM., Berginski ME., Jimenez-Ruiz I., Aksoy BA., Hammerbacher J., et al. Coral: Clear and Customizable Visualization of Human Kinome Data. *Cell Syst* 2018;**7**(3):347-350.e1. Doi: 10.1016/J.CELS.2018.07.001.

2. Salas A., Hernandez-Rocha C., Duijvestein M., Faubion W., McGovern D., Vermeire S., et al. JAK-STAT pathway targeting for the treatment of inflammatory bowel disease. *Nat Rev Gastroenterol Hepatol* 2020;**17**(6):323–37. Doi: 10.1038/S41575-020-0273-0.

3. Garrido-Trigo A., Salas A. Molecular Structure and Function of Janus Kinases: Implications for the Development of Inhibitors. *J Crohns Colitis* 2020;**14**(Supplement_2):S713–24. Doi: 10.1093/ECCO-JCC/JJZ206.

4. Schwartz DM., Kanno Y., Villarino A., Ward M., Gadina M., O’Shea JJ. JAK inhibition as a therapeutic strategy for immune and inflammatory diseases. *Nat Rev Drug Discov* 2017;**16**(12):843–62. Doi: 10.1038/NRD.2017.201.

5. Hu X., li J., Fu M., Zhao X., Wang W. The JAK/STAT signaling pathway: from bench to clinic. *Signal Transduct Target Ther* 2021;**6**(1). Doi: 10.1038/S41392-021-00791-1.

6. Tait Wojno ED., Hunter CA., Stumhofer JS. The Immunobiology of the Interleukin-12 Family: Room for Discovery. *Immunity* 2019;**50**(4):851–70. Doi: 10.1016/J.IMMUNI.2019.03.011.

7. Zlotnik A., Yoshie O. The chemokine superfamily revisited. *Immunity* 2012;**36**(5):705–16. Doi: 10.1016/J.IMMUNI.2012.05.008.

8. Mantovani A., Dinarello CA., Molgora M., Garlanda C. Interleukin-1 and Related Cytokines in the Regulation of Inflammation and Immunity. *Immunity* 2019;**50**(4):778–95. Doi: 10.1016/J.IMMUNI.2019.03.012.

9. Dougan M., Dranoff G., Dougan SK. GM-CSF, IL-3, and IL-5 Family of Cytokines: Regulators of Inflammation. *Immunity* 2019;**50**(4):796–811. Doi: 10.1016/J.IMMUNI.2019.03.022.

10. Murakami M., Kamimura D., Hirano T. Pleiotropy and Specificity: Insights from the Interleukin 6 Family of Cytokines. *Immunity* 2019;**50**(4):812–31. Doi: 10.1016/J.IMMUNI.2019.03.027.

11. Leonard WJ., Lin JX., O’Shea JJ. The γc Family of Cytokines: Basic Biology to Therapeutic Ramifications. *Immunity* 2019;**50**(4):832–50. Doi: 10.1016/J.IMMUNI.2019.03.028.

12. Ouyang W., O’Garra A. IL-10 Family Cytokines IL-10 and IL-22: from Basic Science to Clinical Translation. *Immunity* 2019;**50**(4):871–91. Doi: 10.1016/J.IMMUNI.2019.03.020.

13. McGeachy MJ., Cua DJ., Gaffen SL. The IL-17 Family of Cytokines in Health and Disease. *Immunity* 2019;**50**(4):892–906. Doi: 10.1016/J.IMMUNI.2019.03.021.
