# Supplementary figures and images for: Mucosal kinase activity and inflammatory profiles in inflammatory bowel disease, and in relation to tofacitinib response
Source: J Crohns Colitis. 2025 Sep 23;19(10):jjaf174. doi: 10.1093/ecco-jcc/jjaf174 (PMC12597136; doi:10.1093/ecco-jcc/jjaf174)

# Baseline colonic mucosal cytokine/chemokine levels related to response to tofacitinib

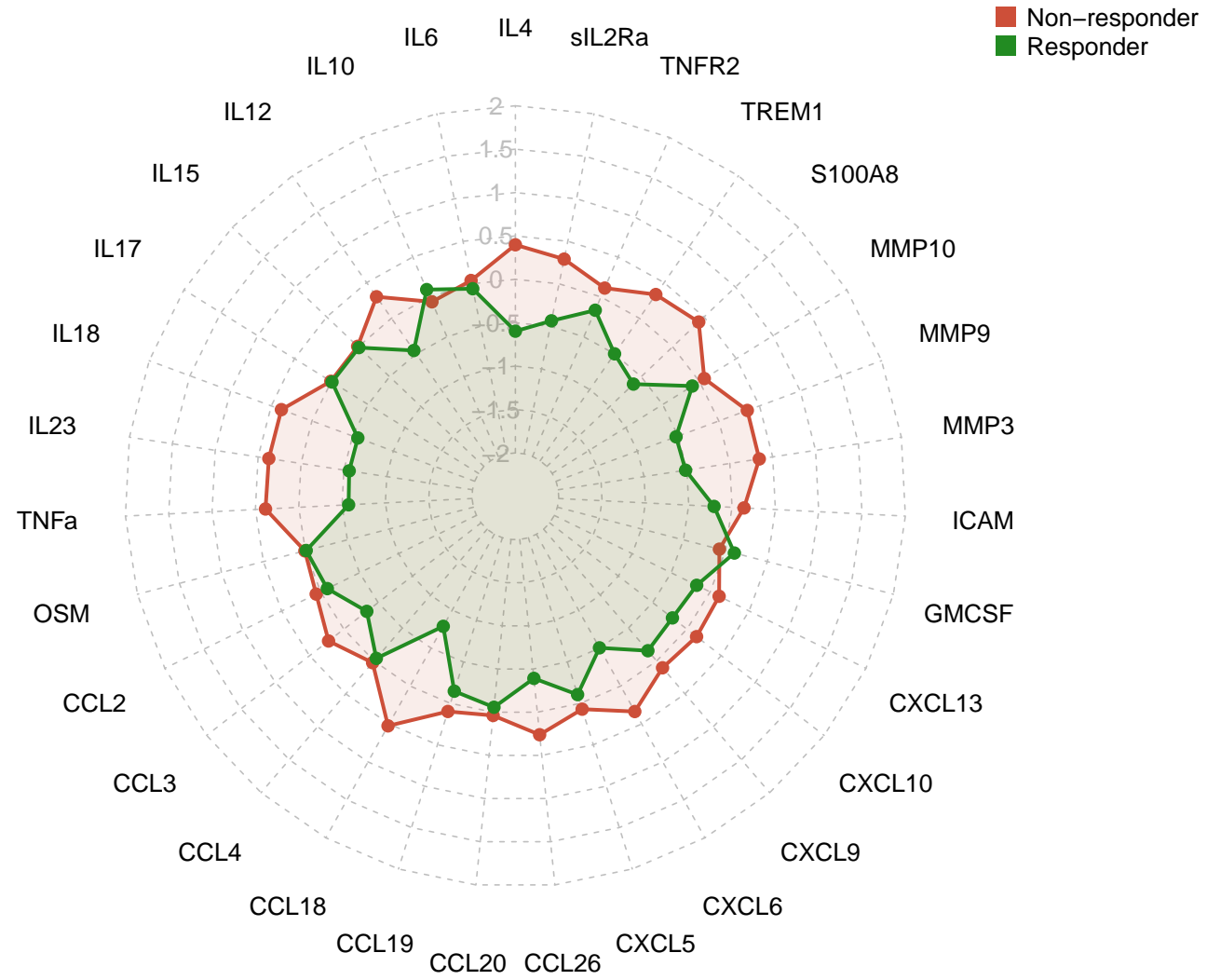

Supplement: jjaf174_Supplementary_Data [file jjaf174_supplementary_data.zip › Supplement Data/Supplementary figure 4.pdf]
